# Coiled-Coil Domain Containing Protein 124 Is a Novel Centrosome and Midbody Protein That Interacts with the Ras-Guanine Nucleotide Exchange Factor 1B and Is Involved in Cytokinesis

**DOI:** 10.1371/journal.pone.0069289

**Published:** 2013-07-19

**Authors:** Pelin Telkoparan, Serap Erkek, Elif Yaman, Hani Alotaibi, Defne Bayık, Uygar H. Tazebay

**Affiliations:** 1 Department of Molecular Biology and Genetics, Faculty of Science, Bilkent University, Ankara, Turkey; 2 Department of Molecular Biology and Genetics, Gebze Institute of Technology, Kocaeli, Turkey; Institut de Génétique et Développement de Rennes, France

## Abstract

Cytokinetic abscission is the cellular process leading to physical separation of two postmitotic sister cells by severing the intercellular bridge. The most noticeable structural component of the intercellular bridge is a transient organelle termed as midbody, localized at a central region marking the site of abscission. Despite its major role in completion of cytokinesis, our understanding of spatiotemporal regulation of midbody assembly is limited. Here, we report the first characterization of coiled-coil domain-containing protein-124 (Ccdc124), a eukaryotic protein conserved from fungi-to-man, which we identified as a novel centrosomal and midbody protein. Knockdown of Ccdc124 in human HeLa cells leads to accumulation of enlarged and multinucleated cells; however, centrosome maturation was not affected. We found that Ccdc124 interacts with the Ras-guanine nucleotide exchange factor 1B (RasGEF1B), establishing a functional link between cytokinesis and activation of localized Rap2 signaling at the midbody. Our data indicate that Ccdc124 is a novel factor operating both for proper progression of late cytokinetic stages in eukaryotes, and for establishment of Rap2 signaling dependent cellular functions proximal to the abscission site.

## Introduction

Centrosomes are microtubule-organizing centers (MTOCs) that play a key role in determining the geometry of microtubule arrays in animal cells. They control and influence cell shape, polarity, motility, spindle formation, as well as chromosome segregation and cell division [1]. Each centrosome comprises a pair of centrioles that are surrounded by an amorphous and dynamic proteinaceous matrix referred to as the pericentriolar material (PCM), which is considered to be the site where microtubule nucleation initiates [2]. Associated with the multifunctional role of this primary MTOC in the cell, the total amount of PCM organized around centrioles (corresponding to centrosome size) and the composition of PCM vary considerably throughout the cell cycle [2]–[4].

Microtubule-nucleating capacities of centrosomes are increased by recruitment of key PCM proteins such as γ-tubulin and gamma-tubulin complex proteins (GCP) forming the γ-tubulin ring complexes (γ-tuRC), which orchestrate cell division-related MTOC activities leading to the formation of spindle asters and correct positioning of the two spindle poles. These cellular activities are required for genetically stable cells as it facilitates proper segregation of the duplicated chromosomes, ultimately resulting in diploid daughter cells [4]–[7].

Recently, a number of efforts aimed to establish both the precise composition of PCM at different stages of cell cycle, and the nature of dynamic networks of molecular interactions that lead to spatiotemporal regulation of PCM assembly. Jakobsen *et al*. [8] have obtained an extensive coverage of human centrosome proteome by using a mass spectrometry-based proteomics approach combined with an antibody based subcellular screen of candidate proteins [8]. In this study, the authors identified 126 known and 40 novel candidate centrosome proteins, of which 22 were validated as novel centrosome components, and 5 turned out to associate preferentially either with mother or daughter centrioles. These analyses also indicated that the majority (60%) of centrosomal proteins contain coiled-coil domain (CCD) type oligomerization motifs [8], [9], as their predominant structural features, which seem to be important for proper centrosome assembly.

Previous studies have indicated the presence of microtubule nucleation-related proteins that are shared between the centrosome and the midbody, which is the central region of the intercellular bridge that is rich in anti-parallel microtubule bundles emanating from the central spindle [10]. These include for instance γ-tubulin, as the midbody is also a secondary MTOC associated with the contractile ring forming at the site of cleavage furrow ingression [1], [11]. Again, another protein with a CCD motif, Cep55, is centrosomal in interphase cells, but dissociates from the centrosome during entry into mitosis [12]–[14]. Precisely at cytokinetic abscission Cep55 is localized to the midbody where it plays an essential role in recruitment of the endosomal sorting complex required for transport (ESCRT) components such as Alix and tumor susceptibility gene 101 (tsg101, an ESCRT-I member), as well as endobrevin (v-SNARE) [15]–[17]. This is then followed by the recruitment of ESCRT-III components via interactions with Alix and Tsg101, resulting in the translocation of CHMP4B to the midbody [18]. According to current models, polymerization of CHMP4B subunits forms series of cortical rings extending away from the midbody, either as continuous helical contractile filaments [18], [19] or to give rise to a second immediately distal CHMP4B pool [20]. This then deforms the intercellular bridge membrane neck into a narrow constriction, and subsequently induces the abscission event.

Recent studies have also identified a number of factors other than Cep55 that similarly localize to centrosomes early in mitosis but then move to the midbody at cytokinesis where they execute essential functions in fission of the daughter cells [10], [16]. Among those proteins, Polo-like-kinase-1 (Plk1) plays critical roles in centrosome maturation and microtubule organization [7], [21], [22]. At the midzone Plk1 also phosphorylates Cep55 on Ser-436, thereby modulating its interaction with Mklp1, a kinesin-like component of centralspindlin. This provides a temporal control of abscission, by inhibiting functions of Cep55 in the midbody at stages earlier than late-anaphase [23].

During our earlier studies on specificities of Ras-guanine nucleotide exchange factor-1 (RasGEF1) family members [24], we obtained preliminary evidences regarding possible interactions between RasGEF1B and a previously uncharacterized protein known as the coiled-coil domain-containing protein 124 (Ccdc124). Here, we report the first characterization of this conserved human protein, Ccdc124, and show that it is a novel component of the centrosome during interphase and at the G2/M transition. During cell division, Ccdc124 relocates to the midbody at telophase and acts as an essential molecular component in cytokinesis. Ccdc124 interacts with RasGEF1B at the midbody where this GEF could activate the small G protein Rap2 [24] at pre-abscission stages. Our data propose a mechanistic link between cytokinesis and Rap signaling that is mainly linked to the formation of cell-cell junctions, regulation of cell-extracellular matrix adhesion, and establishment of cell polarity [25], i.e., molecular processes that must follow cell division while tissues are formed.

## Results

### Molecular Characterization of Ccdc124

The coiled-coil domain (CCD) is a motif that is found in most centrosome proteins [8]. Intrigued by its strict conservation in all eukaryotic genomes from fungi-to-man, we hypothesized that *CCDC124*, a human gene of unknown function encoding a putative CCD containing novel protein, could in fact be involved in centrosome biology. We carried-out a comparative sequence analysis on *CCDC124* which encodes a cDNA that is transcribed from chromosome 19p13.11, consisting of five exons, of which exon 1 is non-coding. BLAST analysis indicated that the protein encoded from this genetic locus shares, for instance, 70% identity/89.1% similarity with its orthologue NP_956859 in the vertebrate model *Danio rerio* (zebrafish), or 50.4% identity/72.6% similarity with Y73E7A.1 in the invertebrate *Caenorhabditis elegans*. *CCDC124* has also orthologues in lower eukaryotes such as the filamentous fungus *Aspergillus nidulans* (AN0879.2; 35.1% identity/58.2% similarity), or the fission yeast *Schizosaccharomyces pombe* (SPBC29A10.12; 33% identity/57.8% similarity), while it is not found in the budding yeast *Saccharomyces cerevisiae*.

**Figure 1:**
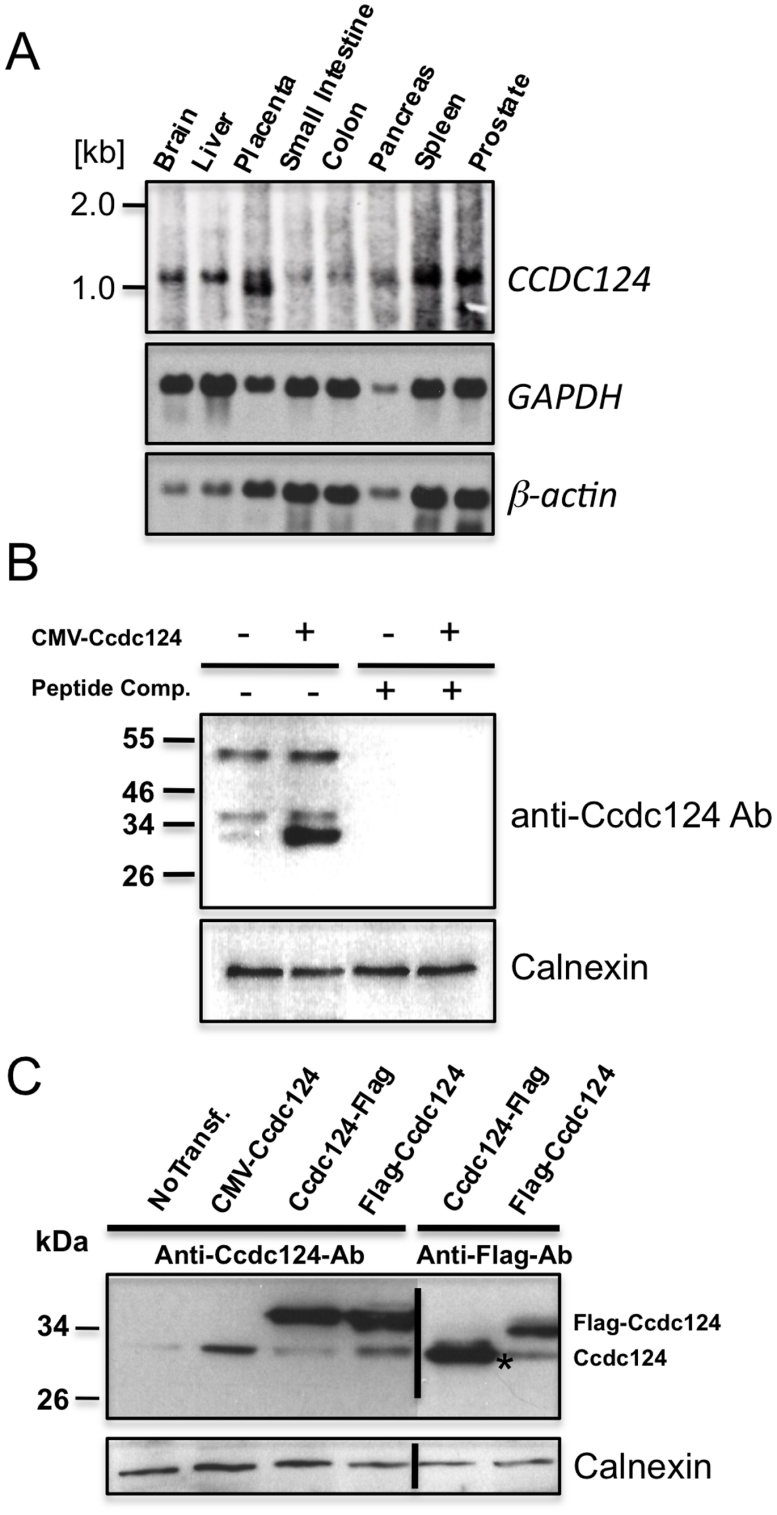
*CCDC124* mRNA is ubiquitously expressed in human tissues, and it encodes a 32 kDa protein. (A) Hybridization of part of the coding region of *CCDC124* to an adult human multiple tissues Northern blot containing 2 µg of polyA-mRNA each lane. A single transcript of ∼1061 nucleotides was detectable in all human tissues analyzed, except the placenta with a second smaller transcript variant. The same blot was rehybridized with probes corresponding to two differentially expressed genes, β-actin and GAPDH, to monitor blotting quality. (**B**) Specific detection of ectopically expressed Ccdc124 by anti-Ccdc124 antibodies. HEK-293 cells, either non-transfected, or transfected with CMV-promoter controlled Ccdc124 were lysed, protein lysates were separated by SDS-PAGE, and immunoblot was performed either with anti-Ccdc124 antibodies alone, or same antibodies pre-incubated with 100 ng of competing peptide epitope corresponding to N-terminus 24mer peptide of Ccdc124. (**C**) Expression of Flag-tagged Ccdc124 protein was specifically detected by the anti-Ccdc124 or with anti-Flag antibodies, as indicated. Asterisk (*) indicates C-terminus flag-tag insertion dependent N-terminus cleaved form of Ccdc124. The expression of calnexin was confirmed in all cell lysates as an equal loading control.

Northern blot analysis have revealed that *CCDC124* is ubiquitously expressed in all tested human tissues, and relatively high levels of expression were detected in the brain, placenta, liver, spleen, and prostate (Fig. 1A). In these analyses, a transcript of ∼1061 nucleotides was detectable in tested organs, in agreement with the predicted size of *CCDC124* mRNA in the NCBI databases (http://genome.ucsc.edu), except in the placenta where we observed a second shorter mRNA species indicative of a transcript variant (Fig. 1A). *CCDC124* cDNA would encode a protein of 223 amino acids with two putative coiled-coil domains between residues 18–82 in the N-terminal half of the protein as detected by the ELM (http://elm.eu.org) and COILS (www.ch.embnet.org/software/COILS_form.html) bioinformatics analysis platforms (Fig. S1). No significant homology to other proteins or domains were found.

We generated a rabbit polyclonal antibody recognizing the peptide corresponding to the N-terminal 24 amino acids of Ccdc124 and characterized its specificity towards Ccdc124 in immunoblots including peptide competition assays (Fig. 1B). We identified Ccdc124 as a ∼32 kDa protein in immunoblots using different protein lysates obtained from Ccdc124 expression vector (CMV-Ccdc124) transfected or untransfected human HEK-293 cells (Figs. 1B–C). Furthermore, when the Ccdc124 ORF was tagged with an N-terminal flag-epitope in plasmid vectors, the antibody also detected the flag-Ccdc124 at the expected size (∼35 kDa; Fig. 1C). When these bands were gel extracted and subjected to peptide analyses by mass-spectrometry, the band of ∼35 kDa were identified as the full-size flag-Ccdc124, suggesting that without the flag epitope *CCDC124* would encode a protein of ∼32 kDa (Pelin Telkoparan, Lars A.T. Meijer, and Uygar H. Tazebay, unpublished results). Surprisingly, anti-flag antibodies failed to detect a similar robust band of ∼35 kDa when the epitope was inserted at the C-terminus, but instead they revealed a band of ∼32 kDa in lysates of cells transfected with vectors expressing Ccdc124-flag (Fig. 1C). This indicated possible proteolytic cleavage of the protein at its N-terminus when flag-epitope is inserted to the C-terminus of Ccdc124. We have not further characterized the proteolytic cleavage of this protein at the molecular level, and we used the more stable N-terminus flag-tagged Ccdc124 expressing vector (flag-Ccdc124) in the rest of our studies.

### Ccdc124 is a Novel Centrosome Protein Relocated to Midbody at Telophase

**Figure 2:**
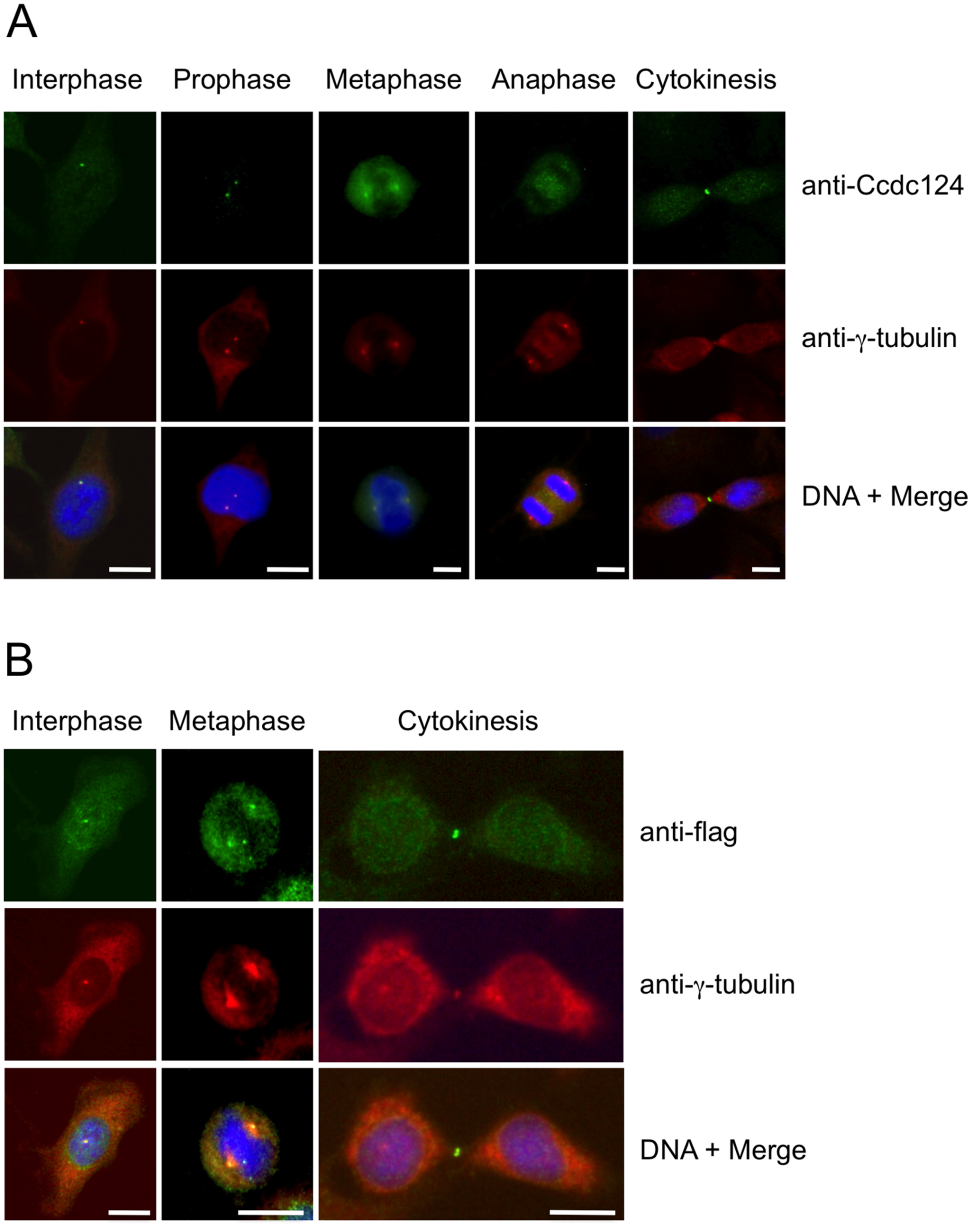
Ccdc124 is present at the centrosome and it concentrates at the midbody in late stages of cytokinesis. (A) HeLa cells were arrested at G2/M phase by sequential double thymidine and nocodazole treatments, then the drug was washed-off, cells were analyzed by immunofluorescence at time points with intervals of 15 minutes, and they were classified according to phases of mitosis, and cytokinesis. Samples of cells were then costained with anti-Ccdc124 and anti-γ-tubulin antibodies, and in interphase and prophase, Ccdc124 was observed as puncta in cells, and it is located in the MTOC area. In metaphase and anaphase cells, Ccdc124 appeared at the spindle poles. Ccdc124 was present at the midzone and at the spindle poles in late anaphase, and concentrated in the midbody during cytokinesis. (**B**) HeLa cells were transfected with the N-ter flag-Ccdc124 vector construct as in Figure 1. 48 hours later samples of cells were subjected to immunofluorescence costainings using anti-flag and anti-γ-tubulin Abs together. Representative micrographs of cells at different stages of cell cycle are given. Bars represent 10 µm.

**Figure 3:**
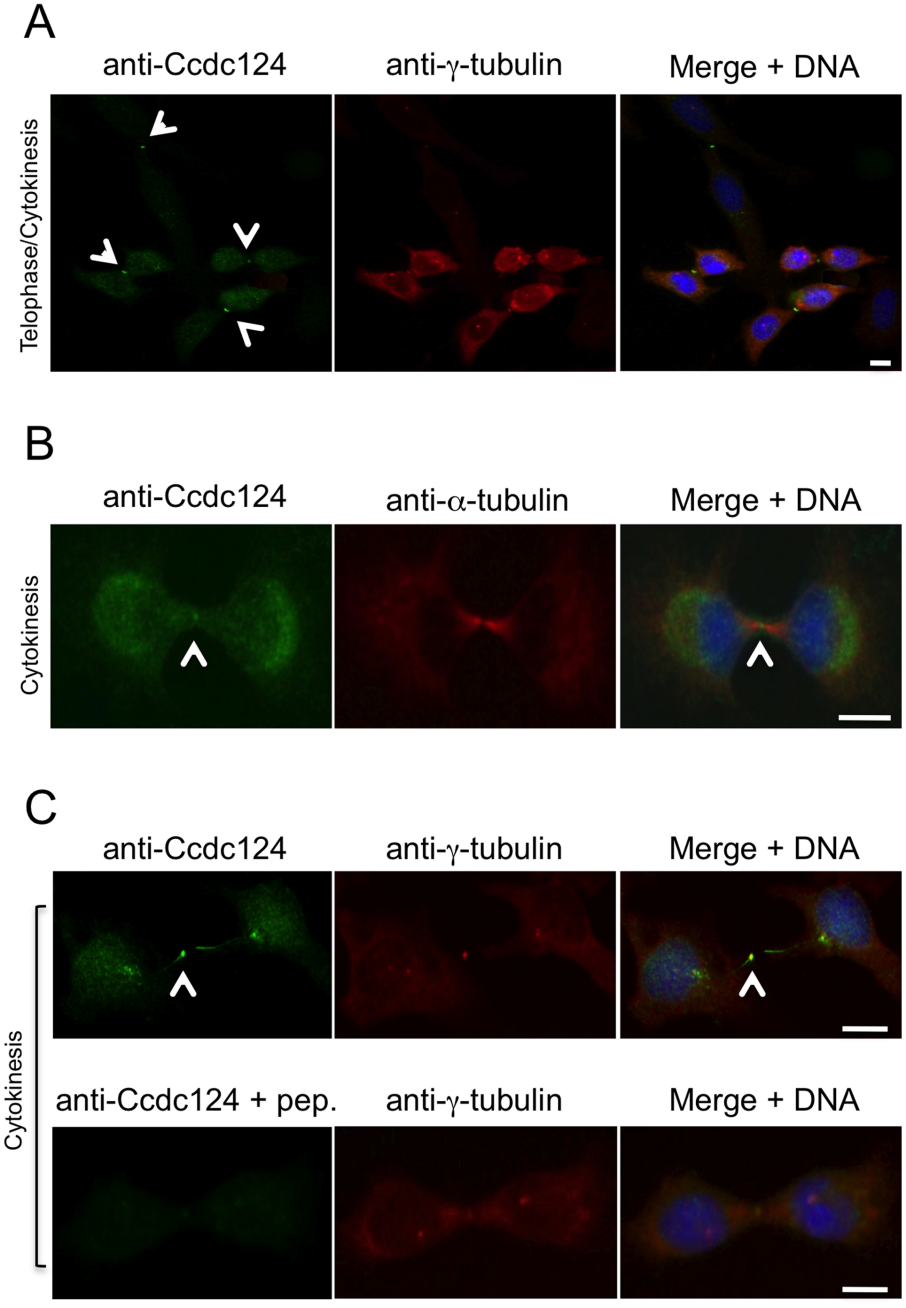
Ccdc124 accumulates at the midbody. HeLa cells were synchronized as described in the legend of Fig. 2, and samples of cells were then stained with anti-Ccdc124 Ab together either with anti-γ-tubulin or with anti-α-tubulin Abs to monitor subcellular positions of centrosomes and the midbody. (**A–B**) At telophase and cytokinesis Ccdc124 is observed as puncta typically associated with the midbody positioned at the middle of intercellular bridge separating daughter cells, as detected in costainings with anti-γ-tubulin and anti-α-tubulin Abs, respectively. (**C**) Peptide competition assays were done by pre-incubating anti-N-ter-Ccdc124 antibody with the corresponding epitope peptide in 200-fold molar excess amounts. Signals generated by Ccdc124 localized at the midbody (shown with arrowhead) were lost in immunofluorescence assays where peptide pre-treated antibodies were used. Bars represent 10 µm.

**Figure 4:**
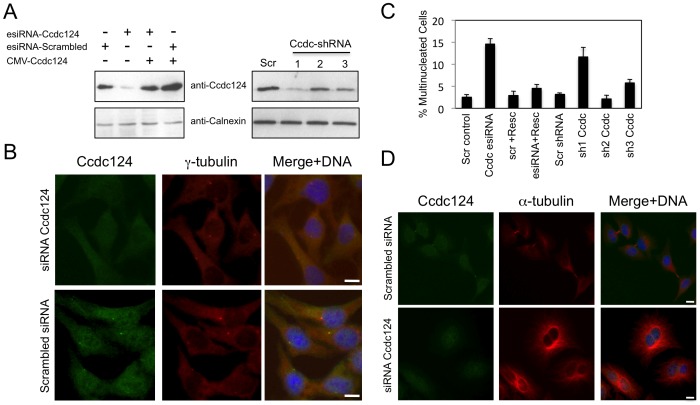
Depletion of Ccdc124 in HeLa cells by RNAi leads to cytokinesis failure. (A) HeLa cells were transfected with either esiRNAs or shRNA vectors (Sh1, Sh2, Sh3) targeting Ccdc124, cell lysates were collected at 48 hrs post-transfection, and immunoblotted with antisera to Ccdc124. Where indicated, Ccdc124 expression vector (CMV-Ccdc124) was cotransfected with gene-specific esiRNAs in order to rescue the cellular effect of Ccdc124 depletion. Scrambled control transfections were indicated (Scr). Calnexin expression was monitored as loading control. (**B**) Immunostainings of endogenous Ccdc124 in cells transfected with Ccdc124-specific esiRNA, or with scrambled control esiRNA were carried out with anti-Ccdc124 Ab. Costainings with γ-tubulin antisera have indicated subcellular positions of MTOCs (**C**) Cells described and analyzed in (A) were scored for bi- and multinucleation (n = 5± SD). (**D**) Representative micrographs of Ccdc124 depleted multinuclear or control esiRNA treated normal dividing cells described in (C). Bars represent 10 µm.

In order to obtain insight into the biological function of Ccdc124, we assessed the subcellular localization of endogenous Ccdc124 by using generated or commercial anti-Ccdc124 antibodies in cellular immunofluorescence assays. When asynchronusly growing HeLa cells were subjected to a preliminary immunofluorescence analysis by using an anti-mid-Ccdc124 antibody recognizing the central part of the protein (between residues 100–150), subcellular dot-like structures reminiscent of centrosomal staining patterns were detected (results not shown, but please see Fig. 2, below). We then synchronized the cells at G2/M stage of the cell cycle by the MT polymerization inhibitor nocodazole following double-thymidine treatments (Fig. S2, see *Methods s1*), and followed cell cycle stage-dependent subcellular localization of endogenous Ccdc124 by immunofluorescence assays using Ccdc124 N-terminal epitope (residues 1 to 24), mid-region, or C-terminal epitope (residues 173 to 223)-specific antibodies. Independent of the Ccdc124 antibody used, these studies further indicated centrosome colocalization of Ccdc124 with γ-tubulin at interphase, prophase, metaphase, and anaphase stages, albeit it was relatively diffused to the pericentrosomal region at anaphase (Fig. 2A). Similar results were obtained when subcellular localization of an N-terminus flag-tagged version of Ccdc124 was monitored by immunofluorescence stainings using anti-flag antibodies on cells transfected with the corresponding vector construct (Fig. 2B). Moreover, endogenous centrosome immunostainings with anti-Ccdc124 Abs were very significantly reduced in response to Ccdc124 depletion by esiRNAs targeting its expression (see below, Fig. 4B), further supporting the notion that Ccdc124 is a novel centrosome protein. Centrosome localization of endogenous Ccdc124 was also observed in Retinal Pigment Epithelial cells (RPE1) containing another centrosomal marker, GFP-Centrin [26] (results not shown). Interestingly, at telophase and in cytokinesis Ccdc124 dissociates from centrosomes and relocalizes to the midzone, subsequently accumulating at the midbody at cytokinesis as assessed by its colocalization with the midzone-specific γ-tubulin (Fig. 2A–B, and Fig. 3A, C), or by its positioning at the midbody marked by the empty mid-space in α-tubulin stainings (Fig. 3B). Immunofluorescence studies with peptide competition assays further indicated that the Ccdc124 signal detected at the midbody was specific, as anti-N-ter-Ccdc124 antibodies pre-treated with the epitope peptide failed to recognize Ccdc124 at the midbody (Fig. 3C).

### Knock-down of CCDC124 by siRNAs Leads to Defects in Cytokinesis

A number of midbody localized proteins were previously shown to be involved in cytokinetic abscission [13], [16], [27]. In order to test a possible role of Ccdc124 during the separation of dividing sister cells, we initially knocked-down *CCDC124* in HeLa cells by separately transfecting them either with esiRNAs or with shRNA vectors specifically targeting this gene. We first monitored knock-down efficiencies by immunoblots that we carried-out using lysates obtained from Ccdc124-specific esiRNA or shRNA vector transfected cells as compared to scrambled esiRNA/shRNA vector transfected controls. We observed approximately 75–80% decrease in Ccdc124 levels in cells that received gene specific esiRNAs as compared to scrambled transfected controls (Fig. 4A). Again, depending on shRNA target sequence, close to 30–65% decrease were detected in Ccdc124 levels in cells separately transfected with three different sequences of shRNAs (Sh1, Sh2, and Sh3), as compared to scrambled controls. In these assays, Ccdc-Sh1 was identified as the vector containing the most potent Ccdc124-targeting shRNA sequence (Fig. 4A) We then analyzed cellular morphologies, centrosome localizations, and midbody functions of asynchronous growing cells that received either Ccdc124 specific esiRNAs, or shRNA vectors targeting the same gene. In Ccdc124-depleted cells interphase centrosomes still formed, as assessed by immunostainings that mark γ-tubulin complexes (Fig. 4B). This indicated that the absence of Ccdc124 does not impair centrosome formation. However, in Ccdc124-depleted cells cytokinesis was remarkably blocked as assessed by the significantly increased percentage of bi- and multinucleated cells (Fig. 4C–D) from 2.5±0.3% in scrambled control esiRNA transfected cases to 14.6±1.5% in Ccdc124-specific esiRNA received asynchronous cultures (Fig. 4C). The percentage of multinucleated cells in Ccdc124-depleted cultures were decreased significantly and nearly restored to normal levels (4.5±0.8%) when Ccdc124 was reintroduced in a strong expression vector (Fig. 4A, C). This indicated that the cytokinesis defect observed with the esiRNA against Ccdc124 was specific. Further supporting this observation, the ratio of multinucleated cells also raised from 3.1±0.2% in scrambled shRNA vector received cells to 11.6±2.7% in cells transfected with Ccdc-Sh1 (Fig. 4B), indicating that defects in cytokinesis was specific to endogenous Ccdc124 depletion in HeLa cells rather than an off-target effect of RNAi.

### Identification and Characterization of Interaction Partners of Ccdc124

In order to identify possible interaction partners of Ccdc124, a human liver cDNA library was screened in a yeast two-hybrid assay system as described in *Materials and Methods*. Colonies with interacting partners were selected, and the corresponding prey fragments were sequenced at their 5′ and 3′ junctions. All 15 positive colonies contained overlapping cDNA sequences belonging to only one gene, RasGEF1B, suggesting that this guanine nucleotide exchange factor (GEF) is a strong candidate as an interaction partner of Ccdc124. RasGEF1B was first identified in zebrafish as a protein expressed in neural cells during late embryogenesis and early larval stages [28]. Furthermore, by using *in vitro* assay systems [24] we previously characterized RasGEF1B as an exchange factor exclusively activating the small G protein Rap2. RasGEF1B was also identified in murine macrophages as a toll-like receptor inducible protein with a subcellular localization in early endosomal vesicles [29].

**Figure 5:**
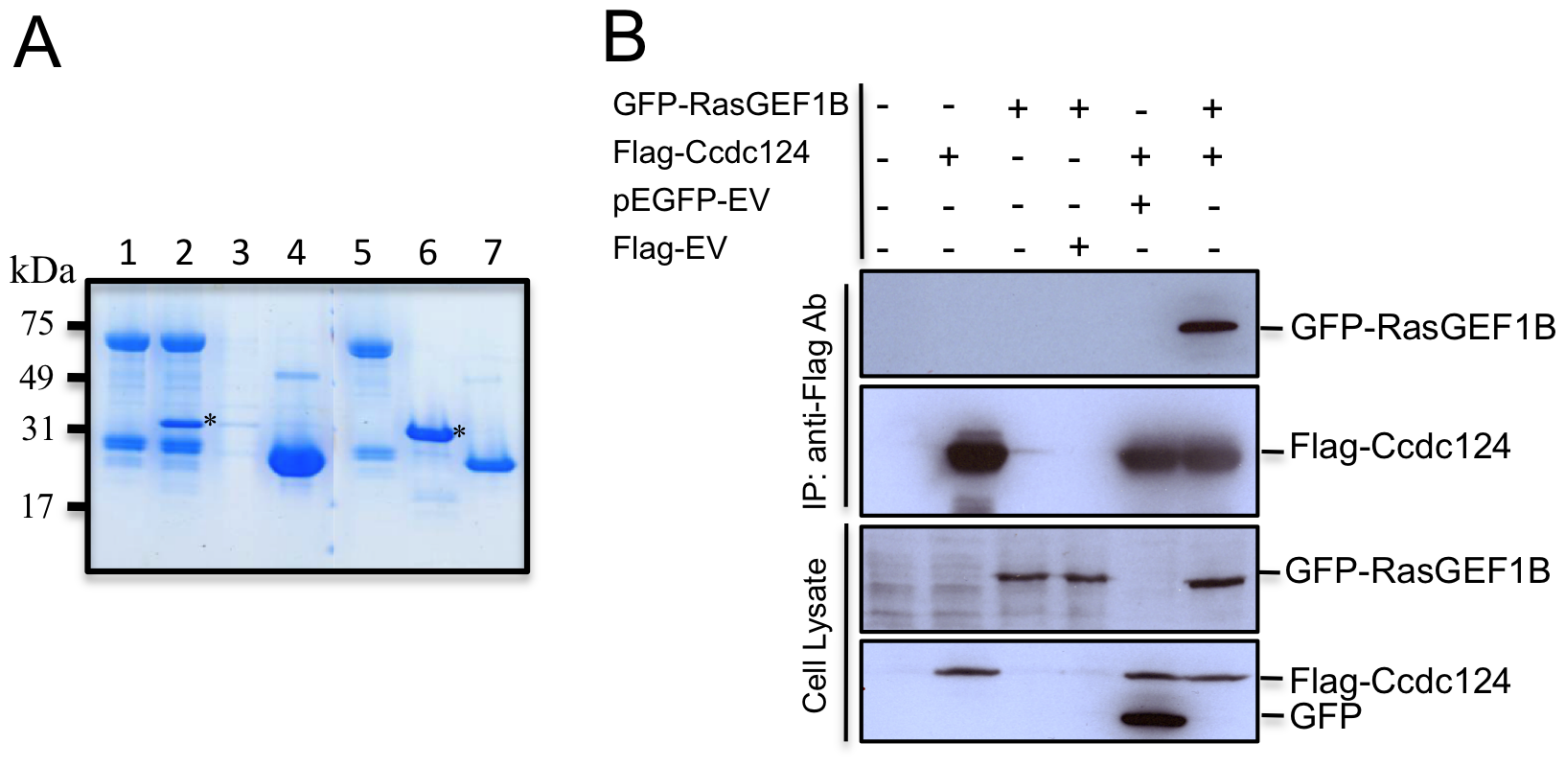
RasGEF1B is an interaction partner of Ccdc124. (A) *In vitro* GST pull-down assay indicating a possible interaction between RasGEF1B and Ccdc124. GST-RasGEF1B protein were immobilized on GSH-beads, followed by incubation with empty PBS buffer control (lane 1), or with bacteria purified His-tagged Ccdc124 (lane 2). As controls, GSH-beads w/o RasGEF1B protein incubated with His-Ccdc124 to monitor the amount of His-Ccdc124 proteins binding to GSH-beads in the absence of a putative interaction partner (lane 3), or GSH-beads immobilized with GST protein and incubated with His-Ccdc124 to monitor interaction capacity of Ccdc124 with GST (lane 4). Lanes 5, 6, 7 are stainings of 100 ng bacteria purified GST-RasGEF1B, His-Ccdc124, and GST proteins, respectively, run in the same gel to monitor their corresponding sizes. Bands corresponding to His-Ccdc124 were marked with asterisks (*). (**B**) HEK-293 cells were either transfected with Flag-Ccdc124 or GFP-RasGEF1B expression vectors alone or with indicated control plasmids, or alternatively they were co-transfected with Flag-Ccdc124 and GFP-RasGEF1B together, followed by immunoprecipitations (IP) on cell lysates using protein-G beads with anti-Flag antibodies. Subsequently, immunoblots were done on IP or cell lysate samples using anti-GFP (monitoring GFP-RasGEF1B) or anti-Flag-HRP (to assess Flag-Ccdc124) antibodies.

**Figure 6:**
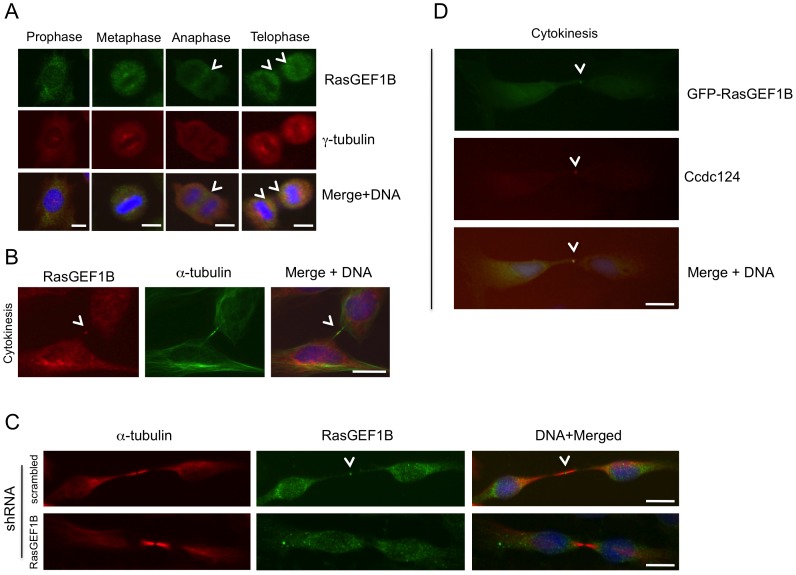
RasGEF1B and Ccdc124 colocalize at the midbody. (A–B) Subcellular localizations of RasGEF1B proteins in synchronously dividing HeLa cells were detected with specific anti-RasGEF1B antibodies. Cell divisions were synchronized as described in the legend of Figure 2, above. Representative immunofluorescence microscopy images of HeLa cells costained with anti-RasGEF1B, and either anti-γ-tubulin (A) or α-tubulin (B) antibodies illustrating the position of MTOCs and the midbody at cytokinesis. Arrowheads show RasGEF1B detected at the midzone and the midbody. (C) Immunofluorescence signals observed at midbody were significantly decreased when endogenous RasGEF1B were depleted by transfections with specific shRNA vectors (Sh-C or Sh-D, Fig. S4) and representative micrographs were shown. (D) HeLa cells transiently transfected with GFP-RasGEF1B were fixed and stained using anti-Ccdc124 Abs. Arrowheads indicate midbody positions of GFP-RasGEF1B, Ccdc124, and their colocalizations at the midbody. Bars represent 10 µm.

Following the yeast two-hybrid assays, we were able to confirm the interaction between Ccdc124 and RasGEF1B, first by *in vitro* GST pull-down methods (Fig. 5A), and then by analyzing their association in mammalian HEK-293 cells transfected with flag-Ccdc124 and GFP-RasGEF1B containing expression vectors (Fig. 5B). Coimmunoprecipitation of GFP-RasGEF1B and flag-Ccdc124 in transfected cells could indicate a functional interaction between these two proteins. In parallel to functional studies to establish cellular roles of Ccdc124 and RasGEF1B, we sought to determine whether the subcellular localizations of these two proteins were comparable throughout the cell cycle. In fact, in a previous study, RasGEF1B was shown to localize in endosomal vesicles when fluorescent protein-fused versions (YFP-RasGEF1B or mRFP-RasGEF1B) were ectopically expressed in asynchronous CHO cells [29]. However, the subcellular localization of RasGEF1B was not previously addressed in cell cycle-synchronized cells. Identification of an endosomal vesicle factor such as RasGEF1B as an interaction partner of centrosomal and/or midbody localized Ccdc124 is particularly interesting, as critical roles of endosomes in physical separation of cells during cytokinetic abscission are well established in recent studies (for a review, see [30]). Subsequently, we generated a rabbit polyclonal antibody recognizing the C-terminal 19 amino acids epitope of the Zebrafish orthologue of RasGEF1B, and by immunoblotting analysis we first established that it also recognizes the human RasGEF1B protein (Fig. S3). Then, by using this antibody, we observed that even though in HeLa cells at interphase and prophase it displays characteristics of previously suggested cytoplasmic/early endosome localization [29], in metaphase cells RasGEF1B was located at a pericentrosomal/centrosomal position, as assessed by its co-localization with γ-tubulin, a subcellular localization similar to that Ccdc124 at metaphase cells (Fig. 6A, compare with Fig. 2).

Again similar to Ccdc124, at telophase and during cytokinesis RasGEF1B was detected at the midzone and in the midbody, and the RasGEF1B immunostaining at the midzone/midbody was sensitive to shRNAs (Fig. S4) targeting the expression of this GEF (Fig. 6B–C), indicating that the immunofluorescence signal was specific to the midbody localized RasGEF1B. Furthermore, during late stages of cytokinesis GFP-tagged RasGEF1B localized to the midbody as described for the endogenous protein (Fig. 6D). Noticeably, in these cell assays GFP-RasGEF1B clearly colocalized with Ccdc124 at the midbody puncta, suggesting that in late-cytokinesis the midbody forms a site of interaction for the two proteins. These results imply a possible role of Ccdc124 in linking cytokinesis to previously uncharacterized RasGEF1B dependent signaling at the midbody. As a note, we observed that bacteria purified Ccdc124 does not functionally interfere with the guanine nucleotide exchange activity of RasGEF1B in *in vitro* reconstituted assay systems (see below, Elif Yaman, Alfred Wittinghofer, and Uygar H. Tazebay, unpublished results), suggesting that Ccdc124 could affect spatial regulation of RasGEF1B, rather than modulating its GEF activity.

### RasGEF1B Stimulates Guanine Nucleotide Exchange of Rap2 in Mammalian Cells in Culture

**Figure 7:**
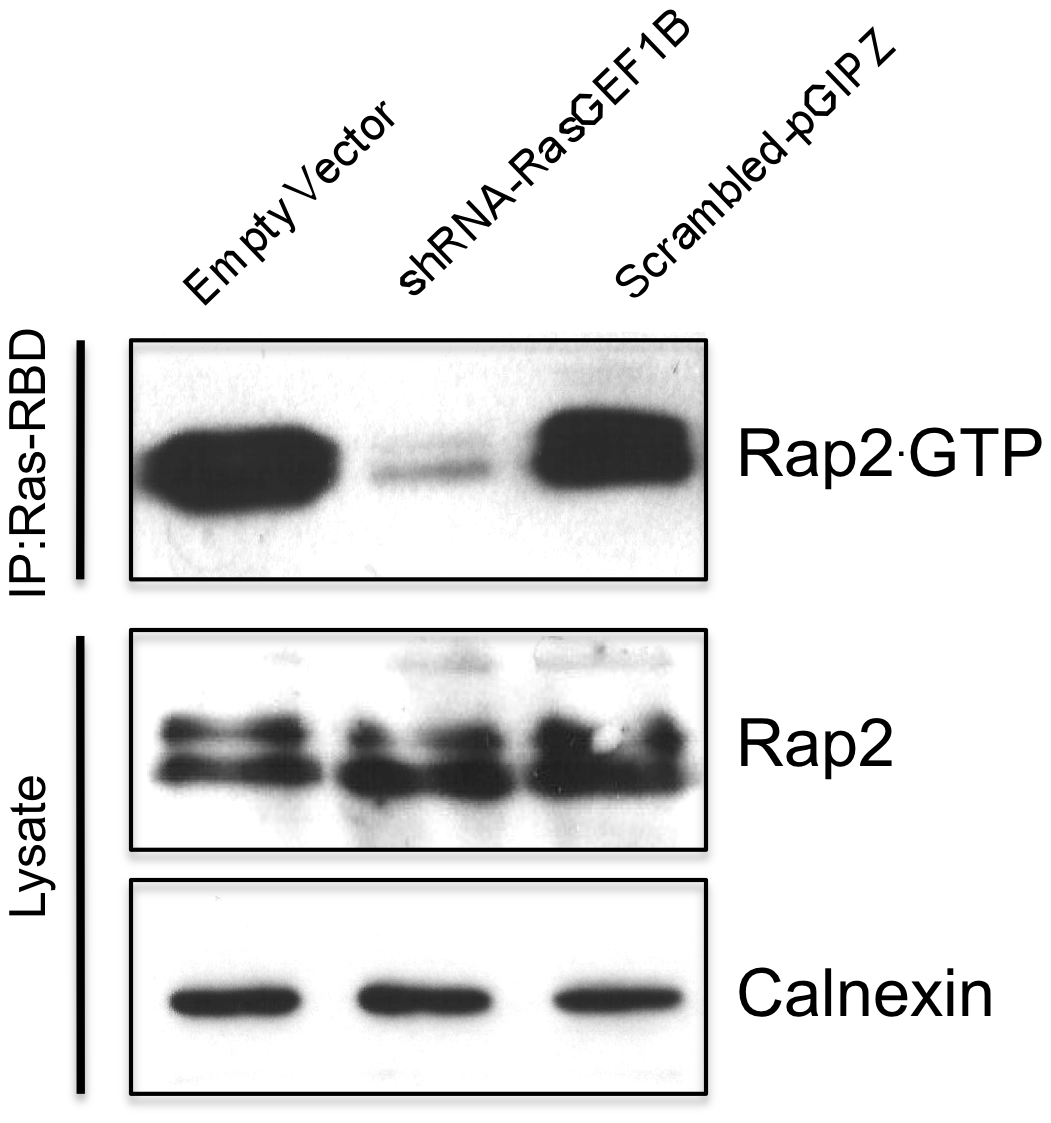
The guanine nucleotide exchange factor RasGEF1B controls cellular Rap2^.^GTP status. HEK-293 cells were transfected either with RasGEF1B specific shRNA expressing vector (Sh-D, Figure S4), scrambled shRNA expressing vector, or empty vector (negative control), 48 hrs later cells were lysed as described at Materials and Methods, they were cleared by centrifugation, and active Rap was precipitated with a glutathione *S*-transferase fusion protein of the Ras-binding domain of RalGDS precoupled to glutathione-Sepharose beads. Rap2 activation assay were carried-out by using anti-Rap2 monoclonal Abs. Results confirmed that shRNA mediated down-regulation of RasGEF1B expression effectively block generation of Rap2**^.^**GTP, although total cellular Rap2 was not affected. Calnexin expression was monitored as loading control.

Activation cycle of small G proteins is regulated by guanine nucleotide exchange factors (GEFs), which induce dissociation of bound GDP and its replacement by the more abundant GTP, and the resulting conformational change allows the binding of effector proteins and thereby stimulation of downstream signaling. Previous functional studies by Yaman *et al*. [24] indicated that under *in vitro* conditions RasGEF1B specifically activates Rap2 by stimulating guanine nucleotide exchange only of this small G protein, whereas it does not activate even its close family member, Rap1, or other members of Ras family. We decided to take these *in vitro* studies to one level up, and confirm the stimulatory effect of RasGEF1B on Rap2 GDP/GTP nucleotide exchange by knocking-down this GEF in HEK-293 cells, followed by assessing GTP-bound active Rap2 (Rap2**^.^**GTP) status in these cells. For this, we used a Rap-activity assay method based on immunoprecipitation of active Rap proteins by the Rap-binding domain of RalGDS (RBD-RalGDS), as previously described [31]. When RasGEF1B is knocked-down to nearly 55% in HEK-293 cells by specific down-regulatory shRNA-containing vectors (Fig. S4), significantly less Rap2**^.^**GTP were present in cells, even though total Rap2 amounts were not affected (Fig. 7). This indicated a functional link between RasGEF1B and Rap2 GTP-binding protein activation in this cell system. These results were consistent with our previous *in vitro* studies that established Rap2 as the sole RasGEF1B activated Ras-family GTP-binding protein [24].

### Active Rap2 GTP-binding Protein is Relocated to the Midbody at Cytokinesis

**Figure 8:**
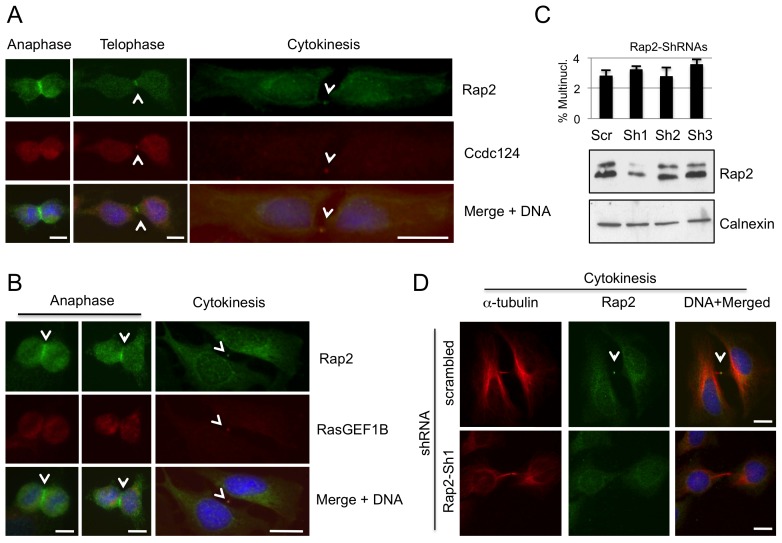
Rap2 colocalize with Ccdc124 and RasGEF1B at the subcellular level. Subcellular localizations of endogenous Rap2 and Ccdc124 or RasGEF1B proteins were studied in HeLa cells by immunofluorescence methods. (**A**) At anaphase, Rap2 was clearly localized at the midzone, while Ccdc124 concentration at the same localization was less pronounced. However, at telophase, both proteins were concentrated at the puncta characterizing the midbody, Rap2 rather surrounding Ccdc124. During cytokinetic abscission, a clear colocalization of both Rap2 and Ccdc124 were observed at the midbody. (**B**) Similar to panel (A), Rap2 translocation to the midzone has started during anaphase. Two representative images of anaphase cells were shown in the corresponding panel. Both Rap2 and RasGEF1B proteins colocalized at the midbody during cytokinetic abscission. Arrowheads either indicate subcellular localization of Rap2 at anaphase and telophase, or they indicate the colocalization of Rap2 with RasGEF1B/Ccdc124 at the midbody during cytokinesis. (**C**) HeLa cells were transfected with shRNA vectors (Sh1, Sh2, Sh3) targeting Rap2, then cell lysates were collected at 48 hrs post-transfection, and immunoblotted with anti-Rap2 Ab. Scrambled control transfection was indicated (Scr). Calnexin expression was monitored as loading control. In parallel experiments, similarly treated cells were immunostained with anti-Rap2 and anti-α-tubulin antibodies, followed by scorings for multinucleation (n = 5± SD), as reported on the graph above the immunoblot. (**D**) Representative micrographs of midbody stage cells depleted in endogenous Rap2 (C). Bars represent 10 µm.

**Figure 9:**
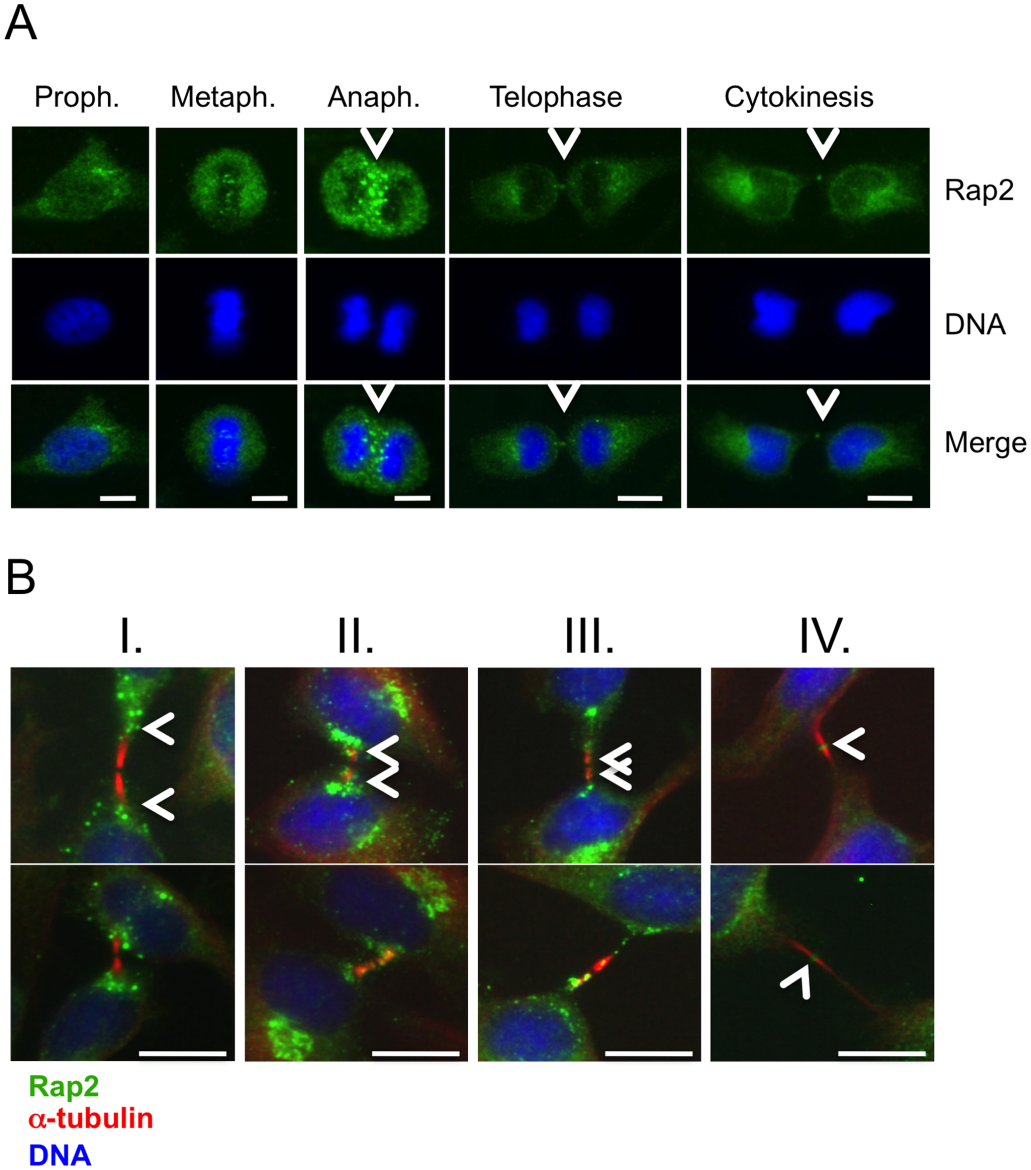
Active endogenous Rap2 relocates to midzone at anaphase, and to midbody during cytokinetic abscission. (A) HeLa cells were arrested at G2/M phase by sequential double thymidine and nocodazole treatments as described in the legend of Figure 2, and they were classified according to phases of mitosis, and cytokinesis. Samples of cells were then stained with anti-Rap2 antibody, and with DAPI to visualize DNA. At anaphase Rap2 was detected at the midzone with staining characteristics reminiscent of endosomes, and at telophase/cytokinesis Rap2 was observed as puncta at the middle of the intercellular bridge, a position typically occupied by midbody associated factors. (**B**) Following synchronization of cells as above, 80 mins. after nocodazole was washed-off samples were taken with four consecutive intervals of 10 minutes (I, II, III, and IV), the last one (IV) corresponding to ∼120 minutes after the drug was removed, and dynamic positioning of Rap2 at the intercellular bridge in respect to α-tubulin was monitored. A time-dependent relocalization of Rap2 from peripheral flanking regions to the midbody was detected. Intercellular bridge localizations of Rap2 were concluded with observations from a sample of ∼50 cells in which over 75% showed similar positioning patterns. Two sets of representative micrographs were displayed. Bars represent 10 µm.

We then decided to assess if RasGEF1B substrate G protein Rap2 was also located in centrosomes and midbody during different stages of cell cycle. Our data indicated that at the midbody the position of Rap2 overlapped both with RasGEF1B and with Ccdc124 in binary comparisons (Fig. 8A, B). Depletion of endogenous Rap2 in HeLa cells by transfecting them with Rap2-specific shRNA vectors have led to the loss of endogenous Rap2 at the midbody (Fig. 8C, D). However, depletion of Rap2 did not result in a significant increase in percentages of multinucleated cells above scrambled controls (Fig. 8C, D). This result could suggest that although Rap2 is located at the midbody, its molecular function(s) at this organelle is(are) not essential for proper completion of cytokinesis. Subsequent immunofluorescence assays using anti-Rap2 antibodies on dividing HeLa cells indicated that Rap2 migrates to the midzone during anaphase/telophase (Fig. 9A, B), which is in contrast to its homologue Rap1 that is non-responsive to RasGEF1B [24] (Fig. S5). We also observed that in synchronously dividing cells 90 mins after they were released from nocodasole, at initial stages of cytokinesis Rap2 associates with microtubules originating at the midzone, and it migrates to the very center of intercellular bridge (boundaries marked with α-tubulin, Fig. 9B), relocating itself to midbody during cytokinetic abscission (Fig. 9A, B).

**Figure 10:**
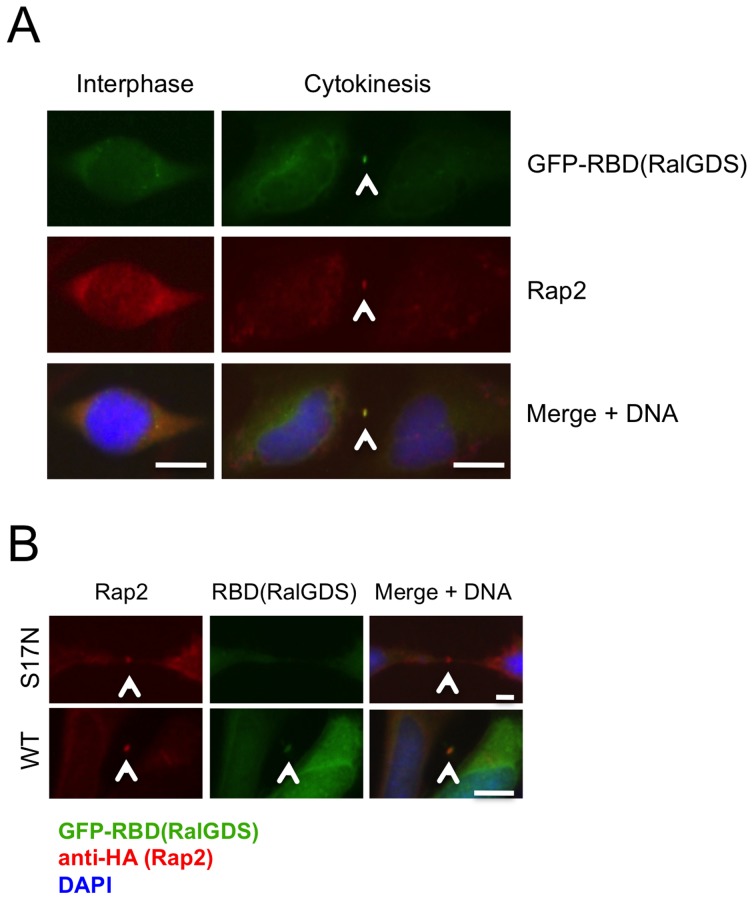
Rap2 effector proteins translocate to the midbody depending on the signal transduction activity of Rap2. (A) HeLa cells were transfected with the vector containing GFP-labeled Rap Binding Domain of RalGDS [GFP-RBD(RalGDS)] which interacts only with the GTP-bound active form of Rap2, and cells were monitored at interphase and at cytokinetic abscission following immunostainings involving anti-Rap2 monoclonal Abs and Alexa568-red labeled anti-mouse secondary Abs. Colocalization of GFP-RBD(RalGDS) with Rap2 indicated that at the midbody Rap2 is in its active (Rap2**^.^**GTP) form. (**B**) HeLa cells were cotransfected with GFP-RBD(RalGDS) either together with HA-Rap2-WT, or with HA-Rap2-S17N (inactive dominant negative form) and their localizations were monitored with anti-HA-epitope Abs. Positioning of GFP-RBD(RalGDS) were assessed by monitoring GFP-signal observed at the midbody. Localization of the GFP-RBD(RalGDS) depended on the presence of active Rap2 in cells. As in (A), at least 50 cells were monitored in each experiment, and representative pictures display Rap2 and GFP-RBD(RalGDS) localizations observed at least ∼90% of cells cotransfected with indicated vectors. Bars represent 10 µm.

We then inquired whether Rap2 located at the midbody was functionally active during cytokinetic abscission, by transfecting HeLa cells with a GFP-labeled recombinant version of the Rap Binding Domain (RBD) of RalGDS referred as GFP-RBD(RalGDS). This GFP-labeled effector protein interacts with Rap2 specifically in the GTP-bound active state of the G protein, and thus serves as a subcellular Rap2 activity reporter [32]. When the signal coming from the GFP-RBD(RalGDS) that interacts with Rap2**^.^**GTP was followed at the subcellular level, at the interphase both endogenous Rap2 and GFP-RBD(RalGDS) were detected to localize diffusely at the cytoplasm with only a partial overlap (Fig. 10A, interphase). However, during cytokinetic abscission, GFP-RBD(RalGDS) very clearly colocalized with Rap2 at the midbody, indicating that Rap2 protein identified at the spindle midzone/midbody is in its GTP-bound active form (Fig. 10A, cytokinesis), and that it may potentially act on its specific effectors at this location. Midbody localization of GFP-labeled RBD(RalGDS) strictly depended on the presence of an active Rap2, as in cells co-transfected with dominant negative version of Rap2 (Rap2-S17N) the reporter protein GFP-RBD(RalGDS) did not relocate with the same efficiency to the midbody during cytokinesis as compared to cells transfected with wild-type Rap2 (Fig. 10B). The endogenous activation of local Rap2 signaling at midbody did not require pretreatment of cells with secondary messengers such as cAMP, diacylglycerol (DAG), or calcium that were previously shown to stimulate Rap signaling through activation of various GEFs [33], [34]. This indicate that the recruitement of RasGEF1B to the midbody could be sufficient for regulation of Rap2 activity, as well as for translocation of Rap2 effectors to this subcellular location.

## Conclusion and Discussion

Previous biochemical studies and mass-spectrometry analyses on purified centrosomes have shown that proteins with CCD (coiled-coil domain) motifs are abundant in PCM [8], [35]. Close to 150 different proteins were identified in the PCM at different cellular stages, about 60% of which contain predicted CCD type oligomerization motifs [8]. In this work, by using molecular and cell biology methods we identified Ccdc124 as a novel component of the centrosome, as in cells at interphase and in mitotic cells up to the late-anaphase/telophase stage it clearly colocalized with two different centrosome markers, such as γ-tubulin and centrin (Fig. 2, and unpublished data). Despite multiple high throughput proteomics analyses targeting centrosome composition in the past, Ccdc124 was not in the list of PCM proteins prior to this study, indicating that combined genetic, cell biology and biochemical approaches are still necessary to identify all the components of PCM which has a remarkably dynamic composition [1], [36]. Conservation of Ccdc124 in lower eukaryotic species without centrosomes, such as *A. nidulans*, or *S. pombe* was not surprising, as also some major centrosome proteins (for instance, γ-tubulin) were common between this organelle and fungal spindle pole bodies [37]. The absence of an orthologue of Ccdc protein in *S. cerevisiae*, however, might indicate that Ccdc124 is not essential for fungi with budding-type of cell division.

Our data revealed that at late-anaphase/telophase stages of cell cycle Ccdc124 changes its subcellular localization: it dissociates from centrosomes, and first relocalizes to the midzone at late-anaphase, and then it accumulates at the midbody puncta at telophase and during cytokinetic abscission (Fig. 2–4). Currently, we do not know what triggers the displacement of Ccdc124 from the centrosome, or its association to the midzone/midbody. In fact, Cep55, a relatively well studied coiled-coil containing centrosome protein that relocates to the midbody and controls cytokinetic abscission, was shown to be a substrate of Plk1 [13]. Phosphorylation of Cep55 by Plk1 on its Ser436 residue is required for its interaction with centralspindlin and ESCRT complex proteins, leading to the recruitment of CHMP4B to the midbody [13], [16]. This is then followed by the assembly of this ESCRT-III component into a series of ring-like structures organizing the abscission site, mainly by bringing the two membranes of the intercellular bridge into close proximity for the scission [20], [38]. Even though in the current study we have not addressed post-translational modifications of Ccdc124, there are several reasons why we anticipate that its functions/stability could be regulated by phosphorylation dependent mechanisms. First, Ccdc124 is identified as a phosphoprotein in our preliminary phosphopeptide analysis by mass-spectrometry methods, and Ser141 residue of Ccdc124 (which is a consensus Plk1 phosphorylation site) was detected as a phosphorylated residue (Pelin Telkoparan, Lars A.T. Meijer, and Uygar H. Tazebay, unpublished results). Second, when we mutated predicted Ser, Thr, or Tyr phosphorylation sites to Ala residues in Ccdc124 by *in vitro* mutagenesis, the Ser121 residue conforming to a Casein Kinase-II phosphorylation consensus site turned out to be essential for the stability of Ccdc124 protein, even though phospho-mimicking mutations S121D, and S121E were normal in terms of protein stability (Fig. S6A, B), indicative of possible phosphorylation dependent regulatory mechanisms operating on Ccdc124.

We found that Ccdc124 depleted cells can still form MTOC, but they undergo cytokinesis failure inducing aneuploidy and generating genomic instability in cultured human cells (Fig. 4). According to current models of cytokinetic abscission, resolution of the membrane connection between two prospective daughter cells requires a concerted action of ESCRT proteins together with the targeting of three main types of recycling endosomes to midbody for an appropriate regulation of cytokinetic abscission (see below) [39], [40]. In an early work, Gromley *et al*, proposed a role for secretory vesicle fusion in the final stages of cytokinetic abscission, as they have shown that the coiled-coil protein centriolin relocates to midbody where, preceding abscission, it interacts with components of vesicle-targeting exocyst complexes and membrane-fusion inducing SNARE components [11]. However, subsequent studies indicated that even though the secretary pathway could contribute to formation of the intracellular bridge membrane, it is rather recycling endosome-dependent mechanisms that make major contributions to spatiotemporal regulation of cytokinetic abscission [40]. Furthermore, other than two Ral family G proteins RalA and RalB, endosome enriched complexes such as Rab35/OCRL and FYVE-CENT/TTC19 that were found on different types of endosomes, were previously shown to enable the completion of the final stages of abscission [41]–[43]. Our data suggested possible links between Ccdc124 and recycling endosomes. First, we identified an endosome localized nucleotide exchange factor RasGEF1B [29] as an interaction partner of the coiled-coil protein Ccdc124 (Fig. 5–6). Second, RasGEF1B activated small G protein Rap2 that we detected at the midzone/midbody, was previously reported to colocalize with Rab11-positive endosomes in *Xenopus* early embryos [44]. Importantly, Rab11-positive recycling endosomes containing the effector protein FIP3 (Rap11 Family of Interacting Protein 3) were previously shown to control the reorganization of the cortical actomyosin network during cytokinetic abscission, as they accumulate at the intercellular space between dividing cells and regulate local actin depolymerization by recruiting p50RhoGAP, and thus contributing to further thinning of the bridge [45].

In this study, we primarily focused on the biological significance of the interaction between Ccdc124 and RasGEF1B, rather than studying mechanistic aspects of it. Yet, our studies on the effect of bacteria purified Ccdc124 on the rate of nucleotide exchange by RasGEF1B on Rap2A in *in vitro* reconstituted assay systems suggested that Ccdc124 does not functionally interfere with RasGEF1B activity (Elif Yaman, Alfred Wittinghofer, Uygar H. Tazebay, unpublished observations). We could hypothesize that rather than modulating its GEF activity, Ccdc124 could recruit RasGEF1B to midzone/midbody where the exchange factor activate its substrate G protein(s). A similar spatiotemporal regulation of Rap1 signaling localized at the plasma membrane by recruitement and translocation of its cAMP responsive GEF, Epac1, through activation of Ezrin-Radixin-Moesin (ERM) complex proteins was previously described [46].

We have shown that both the activator RasGEF1B and its partner G protein Rap2 have identical spatiotemporal subcellular distributions (Fig. 8). This indicated that RasGEF1B could potentially activate GDP/GTP exchange of Rap2 at midbody during late-telophase stage of cell cycle and at cytokinesis. Importantly, we observed the RasGEF1B substrate Rap2, but not its close homologue Rap1, accumulated in vesicular structures proximal to the midzone and at the midbody (Fig. 9A, see panels metaphase to cytokinesis, and Fig. S5). Detection of active Rap2 (Rap2**^.^**GTP) binding reporter protein GFP-RBD(RalGDS) in midbody further proved local activation of Rap2 at midbody puncta during cytokinesis (Fig. 10). This midbody localization of GFP-RBD(RalGDS), was only observed in cells having Rap2-WT, but not its dominant negative form, Rap2-S17N, indicating that midbody localization of Rap2 effectors requires activation of this small G protein (Fig. 10B). Neither cellular depletion of endogenous Rap2 by specific shRNA transfections, nor over-expression of the dominant negative form of Rap2 (S17N) has led to cytokinesis defects, as assessed by normal levels of bi- and multinucleated cells in corresponding cellular assays (Fig. 8, 10). Therefore, in our opinion rather than playing a direct role in cytokinesis, localization of Rap2 at the midbody might serve to modulate and/or functionalize local membrane environment for molecular events following cytokinetic abscission, such as establishment of cell-cell junctions, cell-extracellular matrix adhesions, or polarizations of cells after division of daughter cells fully accomplished. When we consider results obtained in this study altogether, we propose Ccdc124 as a novel factor that links cytokinesis to Rap signaling dependent junction/adhesion or cellular polarization promoting molecular mechanisms, thus bonding different cellular events that must closely follow each other in tissues of live organisms.

## Methods

### Ethics Statement

All cell lines of human origin used in this study were obtained legally either from commercial sources, or they were previously published. Material transfer agreements were duly signed between appropriate offices/persons-in-charge at donating and receiving institutions.

### Cell Culture and Reagents

Hek-293 and HeLa cells were maintained in DMEM and Retinal Pigment Epithelial-1 (RPE1) cells were grown in DMEM/F12 (1∶1) supplemented with 10% FBS and kept at 37°C in a humidified 5% CO2 atmosphere. Cell lines used in this work were either from commercial sources, or described in previous publications. RPE1 cells [26] were obtained from Nurhan Özlü (Koç University, Istanbul), and RPE1 lines stably expressing GFP-Centrin [26], and GFP-Plk1 [47] were gifts from Greenfield Sluder (U. Mass. Medical School, MA), and Prasad Jallepalli (Memorial Sloan-Kettering, NY), respectively. 50 nM of Mission® esiRNA from Sigma-Aldrich (cat. EHU004061) was used for silencing Ccdc124 in HeLa cells by using Oligofectamine (Invitrogen) as transfection reagent. We have also targeted Ccdc124 expression by using the following three shRNA vector constructs prepared in pTRIPZ plasmids (Open Biosystems-Thermo) closely following the procedures described by Paddison *et al*. [48]. Ccdc124 specific shRNA sequences inserted in pTRIPZ vectors were as follows: shRNA #1: 5′-TGCTGTTGACAGTGAGCGACTCGACCAGCTGGAACGTAA|GTAGTGAAGCCACAGATGTACTTACGTTCCAGCTGGTCGAGGTGCCTACTGCCTCGGA-3′; shRNA #2: 5′-TGCTGTTGACAGTGAGCGACGAGACCATCAGCTCAGGGA|GTAGTGAAGCCACAGATGTACTCCCTGAGCTGATGGTCTCGGTGCCTACTGCCTCGGA-3; shRNA #3: 5′-TGCTGTTGACAGTGAGCGACCCAAGAAGTTCCAGGGTGA|GTAGTGAAGCCACAGATGTACTCACCCTGGAACTTCTTGGGCTGCCTACTGCCTCGGA-3. pTRIPZ vector containing scrambled shRNA control were purchased (Open Biosystems-Thermo, cat. no. RHS4750). In experiments to rescue cellular effects of Ccdc124 knock-downs by specific esiRNAs, 40 ng of pCDN3.1-CMV-Ccdc124 plasmids were cotransfected with the above indicated amounts of esiRNAs. In order to knock-down RasGEF1B expression, a set of five plasmids containing shRNA sequences specific for human RasGEF1B gene in pLKO1 vectors, and the corresponding scrambled shRNA control plasmid were purchased (Open Biosystems-Thermo) with the catalog number RHS4533 (TRC Lentiviral shRNAs) and following sequences: 1) Oligo ID: TRCN0000072963, 5′-CCGGGCTGACAGATAGACTCAGATTCTCGAGAATCTGAGTCTATCTGTCAGCTTTTTG-3′, renamed as sh-A; 2) Oligo ID: TRCN0000072964, 5′-CCGGCGGAAACATTTCCCTATGATTCTCGAGAATCATAGGGAAATGTTTCCGTTTTTG-3′, renamed as sh-B; 3) Oligo ID: TRCN0000072965, 5′-CCGGCGGTTATTTATGCATCCGTATCTCGAGATACGGATGCATAAATAACCGTTTTTG-3′, renamed as sh-C; 4) Oligo ID: TRCN0000072966, %’-CCGGGCTCTCTACTTGGCTTCTTATCTCGAGATAAGAAGCCAAGTAGAGAGCTTTTTG-3′, renamed as sh-D; 5) Oligo ID: TRCN0000072967, 5′-CCGGGAAGCACTCATCCAGCACTTACTCGAGTAAGTGCTGGATGAGTGCTTCTTTTTG-3′, renamed as sh-E. Similarly, for decreasing Rap2 expression, a set of three plasmids containing shRNA sequences specific for human Rap2 gene in pGIPZ vectors, together with the corresponding scrambled shRNA control vector were purchased (Open Biosystems-Thermo) with the catalog number RHS4531 (TRC Lentiviral shRNAs) and following sequences: 1) Oligo ID: V2LHS_34663, 5′-TGCTGTTGACAGTGAGCGCACTCAGAACAGGTTATGTAAATAGTGAAGCCACAGATGTATTTACATAACCTGTTCTGAGTTTGCCTACTGCCTCGGA-3′, renamed as sh-1; 2) Oligo ID: V2LHS_34666, 5′-TGCTGTTGACAGTGAGCGAAGAGATATAGTTCACAGTTAATAGTGAAGCCACAGATGTATTAACTGTGAACTATATCTCTGTGCCTACTGCCTCGGA-3′, renamed as sh-2; 3) Oligo ID: V2LHS_34662, 5′-TGCTGTTGACAGTGAGCGCTGACCTTGTGTCACTATTTATTAGTGAAGCCACAGATGTAATAAATAGTGACACAAGGTCAATGCCTACTGCCTCGGA-3′, renamed as sh-3. When indicated, cells were synchronized by a first thymidine block (2,5 mM) for 16 hours, released for 8 hours, and then blocked a second time with thymidine for 16 hours, followed by 200 nM nocodasole treatment/12 hours. Mitotic arrested cells were collected by “mitotic shake-off”, and either they were analyzed directly (0 min.), or recultured for 15, 30, 45, 60, 120, 150, and 180 mins. At the beginning of the experiments cell cycle status of samples were established by FACS analysis as described in Fabbro *et al*. [13] and at each time point cells were processed for immunofluorescence.

### Vector Constructions

pCDNA3.1 was used to generate CMV-promoter controlled Ccdc124 expression vector construct by using EcoRI/BamHI restriction sites and 5′-AAGCTTGAATTCATGCCCAAGAAGTTCCAGGGTGAG-3′ (forward) and 5′-GGTACCGGGATCCTCTTGGGGGCATTGAAGGGCAC-3′ (reverse) primers. Cloning of RasGEF1B into pEGFP-C1 (Clontech) was done by using 5′- GTCGACGAATTCTTAAACTCTGCCTAAGAGGCTCGACC-3′ (forward), and 5′-GTCGACGAATTCTTAAACTCTGCCTAAGAGGCTCGACC-3′ (reverse) primers and XhoI/EcoRI sites. Flag-tagged Ccdc124 expressing vectors were constructed by sub-cloning the gene in p3X-Flag-CMV10 or p3X-Flag-CMV14 vectors (Sigma) by using 5′-CTTGAATTCATGCCCAAGAAGTTCCAGGGTGAG-3′ (forward)/5′-GACCGGGATCCTCTTGGGGGCATTGAAGGGCAC-3′ (reverse) and 5′-CTTGAATTCATGCCCAAGAAGTTCCAGGGTGAG-3′ (forward)/5′-GGATCCTCTAGAGTCCTTGGGGGCATTGAAG-3′ (reverse) primers together with EcoRI/BamHI, and EcoRI/XbaI restriction enzyme sites, respectively. HA-epitope linked versions of Rap2 in Rap2-WT-HA, Rap2-G12V-HA, and Rap2-S17N-HA vectors were kind gifts of Daniel Pak (University of California, Berkeley). GFP-RBD(RalGDS) expression vector was a gift from Johannes L. Bos (University Medical Center Utrecht, the Netherlands).

### Rap Activity Assays

Rap activity assays were carried-out as described previously by van Triest and Bos [31]. Briefly, HEK-293 cells grown in 6-cm plates were lysed in buffer containing 1% NP-40, 150 mM NaCl, 50 mM Tris-HCl (pH 7.4), 10% glycerol, 2 mM MgCl_2_, and protease and phosphatase inhibitors. Lysates were cleared by centrifugation, and active Rap was precipitated with a glutathione S-transferase fusion protein of the Rap-binding domain of RalGDS precoupled to glutathione-Sepharose beads.

### Immunoprecipitation, Immunoblotting and GST-Pull Down Assays

Cells lysed in 50 µl lysis buffer consisting of 50 mM Tris 8.0 Base, 250 mM NaCl, proteinase inhibitor cocktail (Roche), and 1% NP40. Protein concentrations were determined by using Bradford assay. 40 µg of whole cell extracts were denatured in gel loading buffer [50 mM Tris-HCl pH 6.8, 1% SDS, 0.02% bromophenol blue, 10% Glycerol and 5% 2-mercaptoethanol] at 95°C for 5 min, resolved by SDS-PAGE using a 12% gel, and electrotransferred onto PVDF membranes (Millipore). For RasGEF1B/Ccdc124 coimmunoprecipitation experiments, HEK-293 cells were transfected with flag-Ccdc124 and YFP-RasGEF1B vectors. 48 hours later, cells were lysed in NP-40 lysis buffer, flag-tagged agarose beads (A2220, Sigma-Aldrich) were used to precipitate proteins for two hours, followed by a centrifugation for 30 sec. at 8800 rpm, then beads were washed three times with NP-40 lysis buffer. 50 µl sample buffers were added to lysates and incubated at 95°C for 5 min, fractionated by SDS-PAGE for immunoblot analysis and transferred onto PVDF membranes (Millipore). Anti-GFP (G1544, Sigma-Aldrich) and anti-Flag-HRP M2 (A8592, Sigma-Aldrich) were used in Western blot analysis. Blotted proteins were visualized using the ECL detection system (Amersham). For GST pull-down assays, 100 µl 50% slurry (∼50 µl packed) GSH beads were washed 3 times with wash buffer (50 mM Tris HCl pH 7.5, 100 mM NaCl, 3 mM β-Mercaptoethanol). First two vials were immobilized with 200 µg purified GST-RasGEF1B protein, second vial was immobilized with 200 µg GST protein, whereas the fourth vial was incubated with buffer only and rotated at 4°C for 1 hour. The first and the third vials incubated with wash buffer only, second and fourth vials incubated with 500 µg bacteria purified His-Ccdc124 protein and incubated at 4°C for 1 hour by rotating. Beads were washed 10 times with wash buffer, and samples were eluted with 40 µl 4xSDS loading buffer, boiled for 5 min and loaded on an SDS gel. For the control of protein sizes samples from a 10 µg/µl stock of each protein were loaded to the same gel. The gel then stained with commassie blue and destained with water.

### Generation of Polyclonal Antibodies

3 mg of N-terminus 24mer peptide MPKKFQGENTKSAAARARRAEAK-[C]-amide of human Ccdc124, and [C]-NNMEKDR-W-KSLRSSLLNRT peptide corresponding to partly conserved C-terminus of the zebra fish homologue of RasGEF1B (*Danio rerio* RasGEF domain family member 1Ba, NCBI accession number: NM_199829) were coupled to KLH through their inserted cysteine residues via MBS, and Both peptides were used in rabbit immunizations at the Cambridge Research Biochemicals (Cambridge, United Kingdoms). 10 mg of each peptide affinity column purified (Glycine and TEA eluates) antibodies and pre-immune control sera were then received for use in molecular biology research.

### Immunofluorescence

HeLa cells on cover slips were fixed by ice cold 100% Methanol for 10 min at −20°C, washed three times with PBS-T; blocked in 2% BSA for 1 hour and incubated 1 hour with primary antibodies, washed again three times with PBS-T and incubated for 1 hour with suitable secondary antibodies. Sources of antibodies: Plk1 a gift from Nurhan Özlü, Koç University, Istanbul; monoclonal (ab17057), or polyclonal (ab109777) Abs were purchased from Abcam; anti-RasGEF1B-C-ter epitope Ab and anti-Ccdc124-N-ter epitope Ab were custom produced by Cambridge Research Biochemicals-UK (see above), Ccdc124 middle-epitope specific (A-301-835A) or C-ter epitope specific (A-301-834A) Abs were from BETHYL Laboratories; anti-γ-tubulin were either from Abcam (ab11316), or from BioLegend (rabbit polyclonal cat.620902); anti-α-tubulin were from Santa Cruz (SC-5286); rabbit anti-flag-Ab was from Sigma (F-7425); rabbit anti-Rap2 polyclonal was purchased from Genetex (GTX108831); mouse anti-Rap2 monoclonal was from BD Biosciences (610215), DNA dye (DAPI) was from Invitrogene (P36931); and Alexa Fluor 488, or 568 labeled anti-rabbit, and anti-mouse secondary antibodies were purchased from Invitrogene.

### Fluorescent Microscopy and Imaging

Fluorescent images were done by Zeiss Imager-A1 microscope with a Zeiss Acroplan 40X objective. Images were captured on the Zeiss Axia Cam MRc 5-CCD camera for fixed samples. Microscope control and image processing were done through Axiovision version 4.6 software program (Universal Imaging).

### Northern Blot Analysis

Northern blotting was performed on commercially obtained “FirstChoice Northern Human Blot-1 and Blot-2 Membranes” (Ambion) which contained 2 µg poly(A)-RNA from indicated human organs on each lane. Donor information is available at the suppliers (Ambion) documents; human derived materials have been prepared in the mentioned company from tissues obtained with consent from a fully informed donor, or a member of the donor’s family, as certified by the company. DNA probes used in hybridizations were as follows: ApaI-XbaI DNA fragment (381 bp) of *CCDC124* cut-out from Flag-Ccdc124 plasmid vector, GAPDH fragment (408 bp) obtained after PCR amplification of a HeLa cell cDNA library by using forward primer 5′-GGCTGAGAACGGGAAGCTTGTCAT-3′ and reverse primer 5′-CAGCCTTCTCCATGGTGGTGAAGA-3′ as amplification primers, and β-actin fragment (539 bp) obtained after PCR amplification of the same cDNA library by using forward 5′- GATGACCCAGATCATGTTTG-3′ and reverse 5′- CATGGAGGAGCCGCCAGACAGC-3′ primers in PCR amplifications in the following conditions: 5 min initial denaturation at 95°C, 35 cycles of 30 sec denaturation at 95°C, 30 sec primer annealing at 60°C and 30 sec extension at 72°C, and a 5 min final extension at 72°C. Then, DNA templates corresponding to expected band sizes were isolated from agarose gels, labeled by North-2-South Biotin random prime labeling kit (Pierce). Nucleic acids hybridization and detection were done by North-2-South Chemiluminescent hybridization and detection kit (Pierce). Resulting blots were exposed to autoradiography films (Kodak).

### Identification of CCDC124-interacting Proteins by using the Yeast Two-hybrid (Y2H) Screening Method

Bait cloning and Y2H screening were performed by Hybrigenics, S.A., Paris, France (http://www.hybrigenics.com). Human CCDC124 cDNA (encoding 223 a.a protein) was PCR-amplified and cloned in a LexA C-terminal fusion vector. The bait construct was checked by sequencing the entire insert, and was subsequently transformed in the L40ΔGAL4 yeast strain [49]. Then, a human liver cDNA library containing ten million independent fragments were transformed into the Y187 yeast strain, which was used for mating. The screen was performed in conditions ensuring a minimum of 50 million interactions tested, in order to cover five times the primary complexity of the yeast-transformed cDNA library. After selection on medium lacking leucine, tryptophane, and histidine, 15 positive clones were picked, and the corresponding prey fragments were sequenced at their 5′ and 3′ junctions. Sequences were contigued as described previously, and then they were compared to the latest release of the GenBank database using BLASTN [50]. A Predicted Biological Score (PBS) was attributed to assess the reliability of each interaction, as described previously [51].

## Supporting Information

Figure S1 **Ccdc124 contains two main coiled-coil domains at its N-terminal part.** Schematic representation of the coiled-coil prediction of Ccdc124 is presented. The graph was obtained by the COILS (www.ch.embnet.org/software/COILS_form.html) bioinformatics analysis platform.
(TIFF)

Figure S2 **Double-thymidine and nocodazole treatments synchronized HeLa cells at G2/M phase of cell cycle.** HeLa cells were treated with thymidine and MT polymerization inhibitor drug nocodazole as indicated in *Methods*. 1×10^6^ unsynchronized **(A)** or synchronized **(B)** cells were collected as samples, and resuspended in 0.3 ml of PBS buffer. Cells were fixed by addition of 0.7 ml cold ethanol (70%), left on ice for 1 hr, and then washed and resuspended in 0.25 ml of PBS in which it is treated with 0.5 mg/ml RNAse-A for 1 hr at 37°C. Cellular DNA is then stained with 10 µg/ml propidium iodide (PI) solution, and cytometric analysis was performed by FACS at 488 nm. Percentages of cells in each sample at various stages of the cell cycle are indicated below each panel **(A–B)**.
(TIFF)

Figure S3 **Polyclonal Anti-RasGEF1B antibody raised against zebrafish homologue of RasGEF1B cross-reacts strongly with human RasGEF1B.** HEK-293 cells were transfected with mCherry-labeled human RasGEF1B or YFP-labeled human RasGEF1B expression vectors (mCherry-RasGEF1B and YFP-RasGEF1B, respectively), after 48 hours cells were lysed, proteins were separated by SDS-PAGE, and immunoblot was performed with anti-RasGEF1B antibody alone (1 µg at 1∶1000 dilution), and then the membrane was stripped and sequentially reprobed first with the same antibody pre-incubated with 100 ng of competing 20mer peptide epitope [C]-NNMEKDR-W-KSLRSSLLNRT corresponding to C-terminus of ZF-RasGEF1B, and then with anti-GFP antibody recognizing YFP as its epitope in YFP-RasGEF1B. Calnexin expression was monitored as loading control.
(TIFF)

Figure S4 **Screening of RasGEF1B specific shRNA plasmids to monitor their down-regulatory capacities.** HEK-293 cells were transfected with RasGEF1B shRNA plasmids described in Materials and MethodsS1. 48 hours after transfections, cells were lysed and proteins were separated by SDS-PAGE. Immunoblot was performed with custom made anti-RasGEF1B antibody. Image-J software program were used to obtain densitometric readings of band intensities corrected by calnexin values, and these were indicated below each band. RasGEF1B specific shRNA expressing vector renamed as sh-D (see *Materials and Methods S1*) was selected to carry-out experiments described in Results. Calnexin expression was monitored as loading control.
(TIFF)

Figure S5 **Endogenous Rap1 does not relocate to the midzone/midbody during cytokinetic abscission.** HeLa cells were arrested at G2/M phase by sequential double thymidine and nocodazole treatments as described in the legend of Figure 2, and they were classified according to phases of mitosis, and cytokinesis. Samples of cells were then costained with anti-Rap1 and anti-α-tubulin antibodies, which were used to monitor intercellular bridge and the space containing midbody complexes. DAPI staining was used to visualize DNA. Bars represent 10 µm.
(TIFF)

Figure S6 **Mutating the consensus CK2 phosphorylation site Ser122 to Ala leads to compromised stability of Ccdc124.** **(A–B)** HEK-293 cells were transfected either with HA-tagged wild-type Ccdc124 expression vector, or with similar vectors carrying indicated mutations on Figures, and stabilities of mutants proteins were monitored by immunoblots using anti-Ccdc124 antibodies recognizing the N-terminus of the protein. Only one CK2 phosphorylation consensus site (Ser122) turned out to be essential for the stability of Ccdc124 protein as S122A mutants were cleaved at their C-terminus, whereas phospho-mimicking mutations S121D, and S121E were normal in terms of Ccdc124 stability (B). Calnexin expressions were monitored as loading control.
(TIFF)

Methods S1 (DOCX)
