# Supplementary figures and images for: Coiled-Coil Domain Containing Protein 124 Is a Novel Centrosome and Midbody Protein That Interacts with the Ras-Guanine Nucleotide Exchange Factor 1B and Is Involved in Cytokinesis
Source: PLoS One. 2013 Jul 19;8(7):e69289. doi: 10.1371/journal.pone.0069289 (PMC3716640; doi:10.1371/journal.pone.0069289)

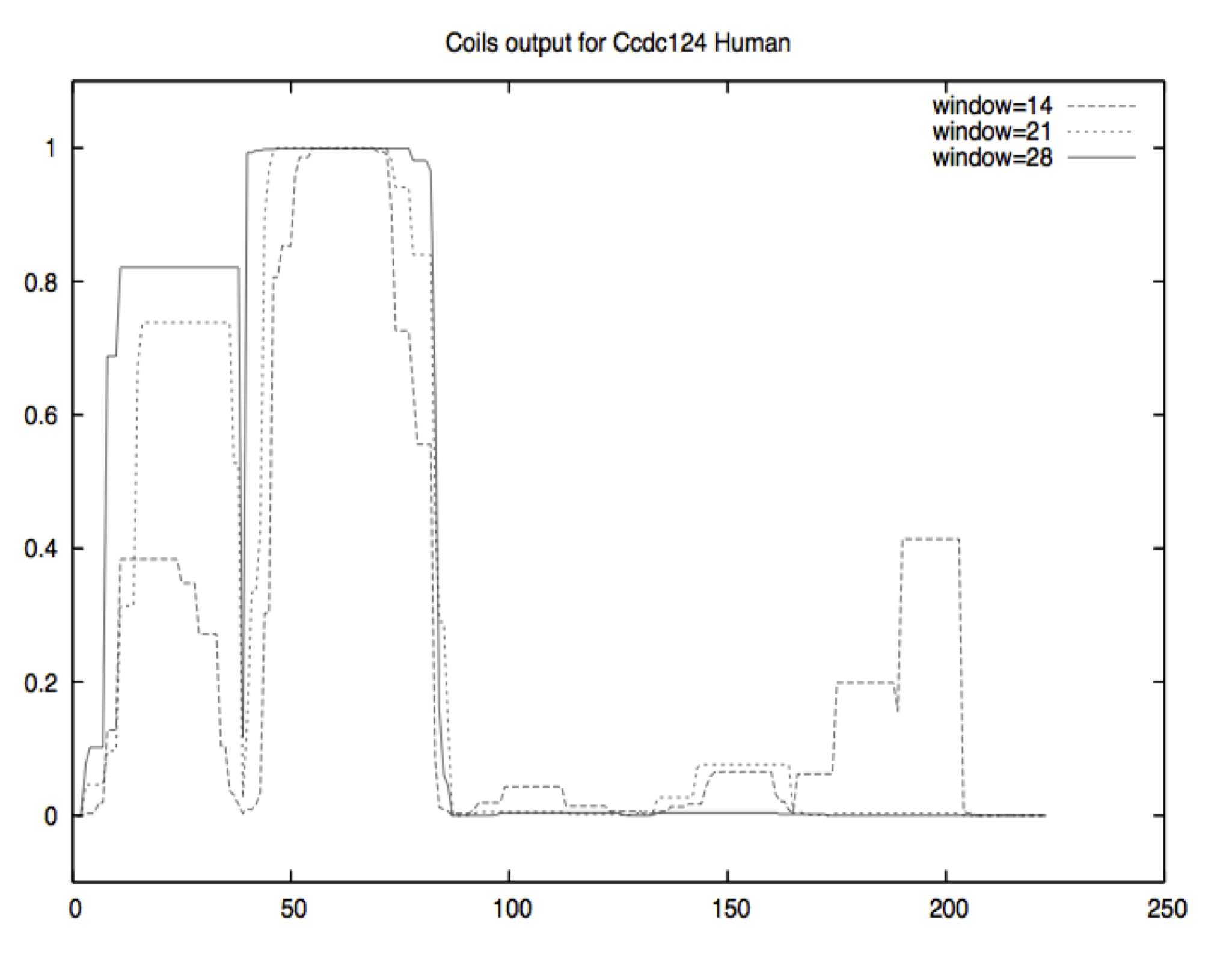

Supplement: Figure S1 — Ccdc124 contains two main coiled-coil domains at its N-terminal part. Schematic representation of the coiled-coil prediction of Ccdc124 is presented. The graph was obtained by the COILS (www.ch.embnet.org/software/COILS_form.html) bioinformatics analysis platform. (TIFF) [file pone.0069289.s001.tiff]

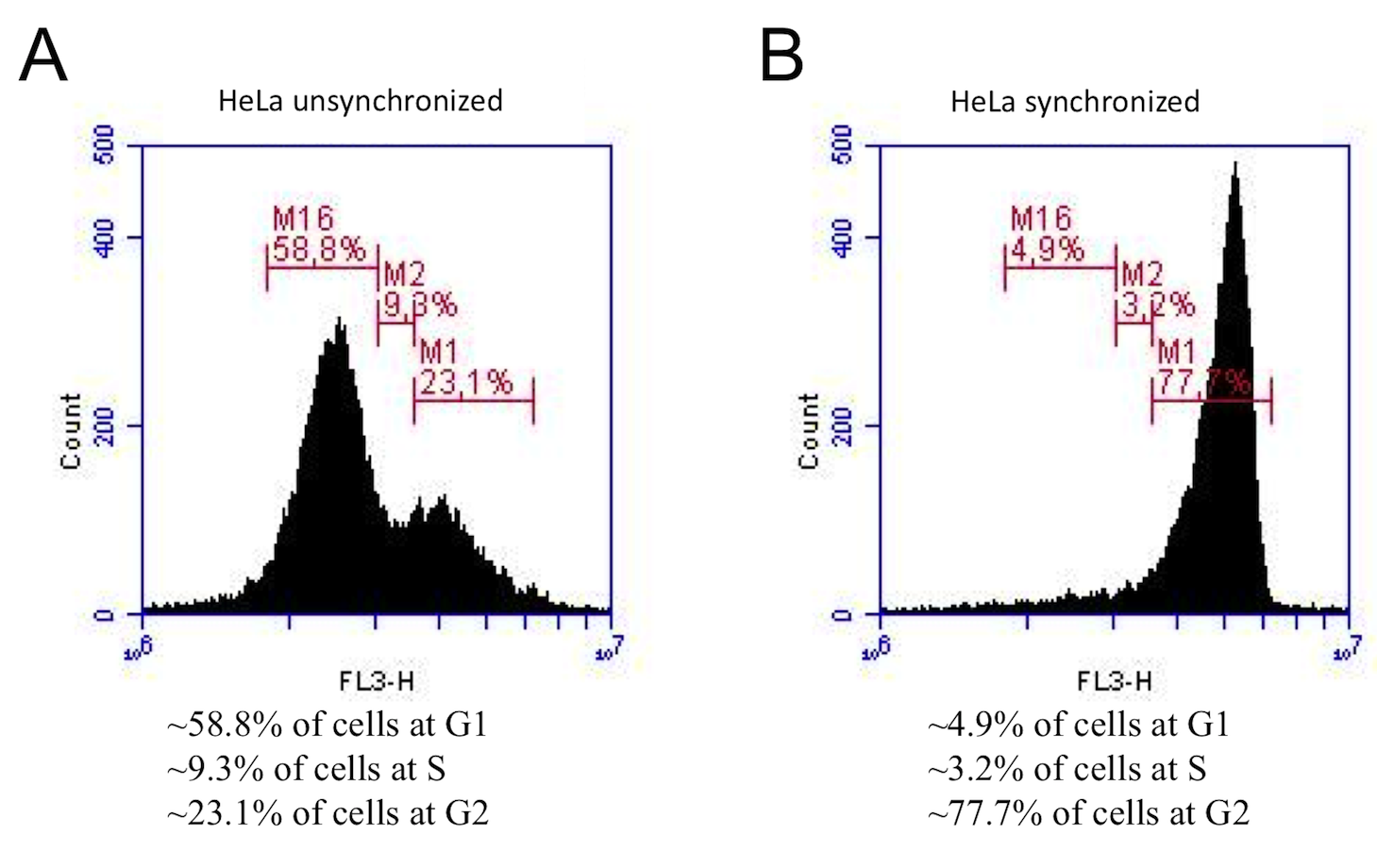

Supplement: Figure S2 — Double-thymidine and nocodazole treatments synchronized HeLa cells at G2/M phase of cell cycle. HeLa cells were treated with thymidine and MT polymerization inhibitor drug nocodazole as indicated in Methods. 1×10^6^ unsynchronized (A) or synchronized (B) cells were collected as samples, and resuspended in 0.3 ml of PBS buffer. Cells were fixed by addition of 0.7 ml cold ethanol (70%), left on ice for 1 hr, and then washed and resuspended in 0.25 ml of PBS in which it is treated with 0.5 mg/ml RNAse-A for 1 hr at 37°C. Cellular DNA is then stained with 10 µg/ml propidium iodide (PI) solution, and cytometric analysis was performed by FACS at 488 nm. Percentages of cells in each sample at various stages of the cell cycle are indicated below each panel (A–B). (TIFF) [file pone.0069289.s002.tiff]

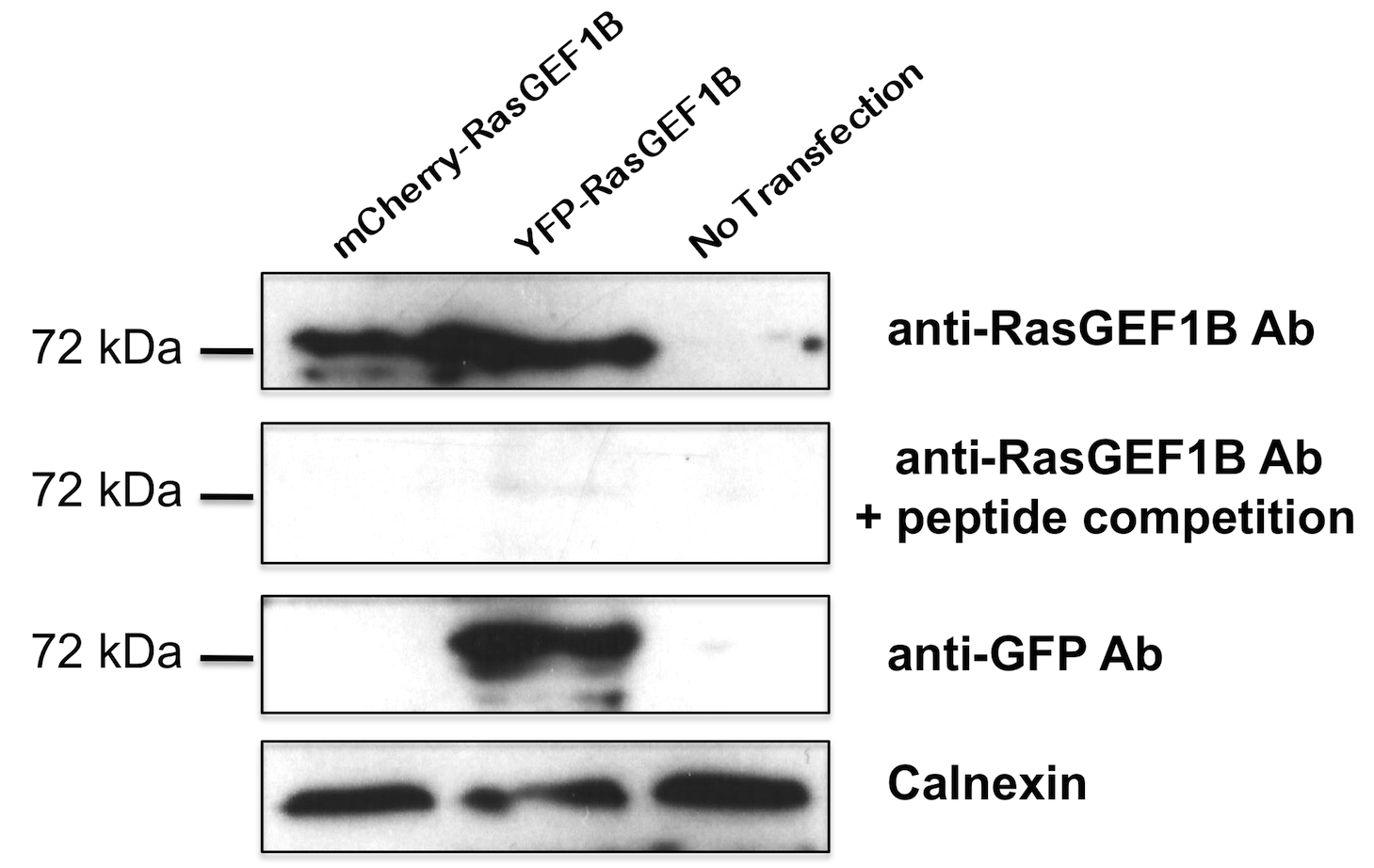

Supplement: Figure S3 — Polyclonal Anti-RasGEF1B antibody raised against zebrafish homologue of RasGEF1B cross-reacts strongly with human RasGEF1B. HEK-293 cells were transfected with mCherry-labeled human RasGEF1B or YFP-labeled human RasGEF1B expression vectors (mCherry-RasGEF1B and YFP-RasGEF1B, respectively), after 48 hours cells were lysed, proteins were separated by SDS-PAGE, and immunoblot was performed with anti-RasGEF1B antibody alone (1 µg at 1∶1000 dilution), and then the membrane was stripped and sequentially reprobed first with the same antibody pre-incubated with 100 ng of competing 20mer peptide epitope [C]-NNMEKDR-W-KSLRSSLLNRT corresponding to C-terminus of ZF-RasGEF1B, and then with anti-GFP antibody recognizing YFP as its epitope in YFP-RasGEF1B. Calnexin expression was monitored as loading control. (TIFF) [file pone.0069289.s003.tiff]

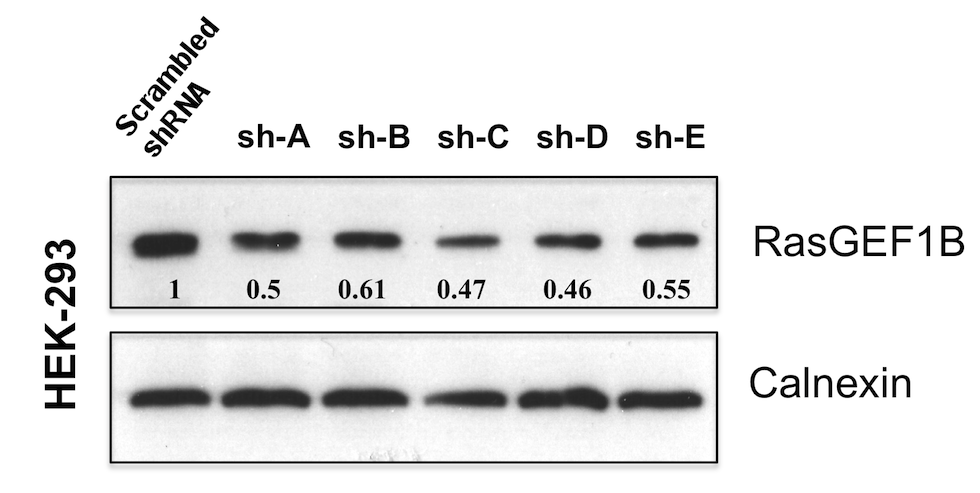

Supplement: Figure S4 — Screening of RasGEF1B specific shRNA plasmids to monitor their down-regulatory capacities. HEK-293 cells were transfected with RasGEF1B shRNA plasmids described in Materials and MethodsS1. 48 hours after transfections, cells were lysed and proteins were separated by SDS-PAGE. Immunoblot was performed with custom made anti-RasGEF1B antibody. Image-J software program were used to obtain densitometric readings of band intensities corrected by calnexin values, and these were indicated below each band. RasGEF1B specific shRNA expressing vector renamed as sh-D (see Materials and Methods S1) was selected to carry-out experiments described in Results. Calnexin expression was monitored as loading control. (TIFF) [file pone.0069289.s004.tiff]

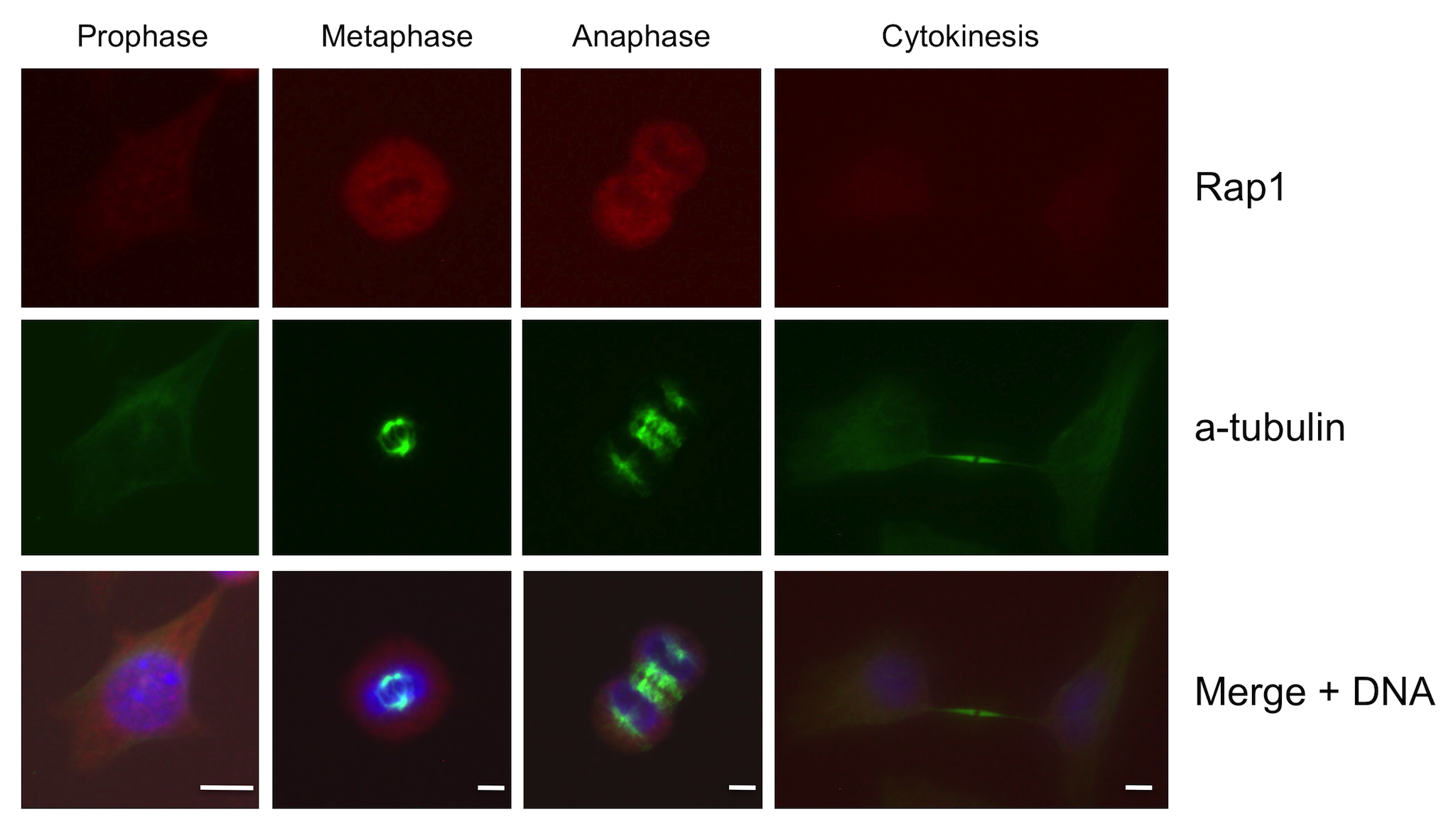

Supplement: Figure S5 — Endogenous Rap1 does not relocate to the midzone/midbody during cytokinetic abscission. HeLa cells were arrested at G2/M phase by sequential double thymidine and nocodazole treatments as described in the legend of Figure 2, and they were classified according to phases of mitosis, and cytokinesis. Samples of cells were then costained with anti-Rap1 and anti-α-tubulin antibodies, which were used to monitor intercellular bridge and the space containing midbody complexes. DAPI staining was used to visualize DNA. Bars represent 10 µm. (TIFF) [file pone.0069289.s005.tiff]

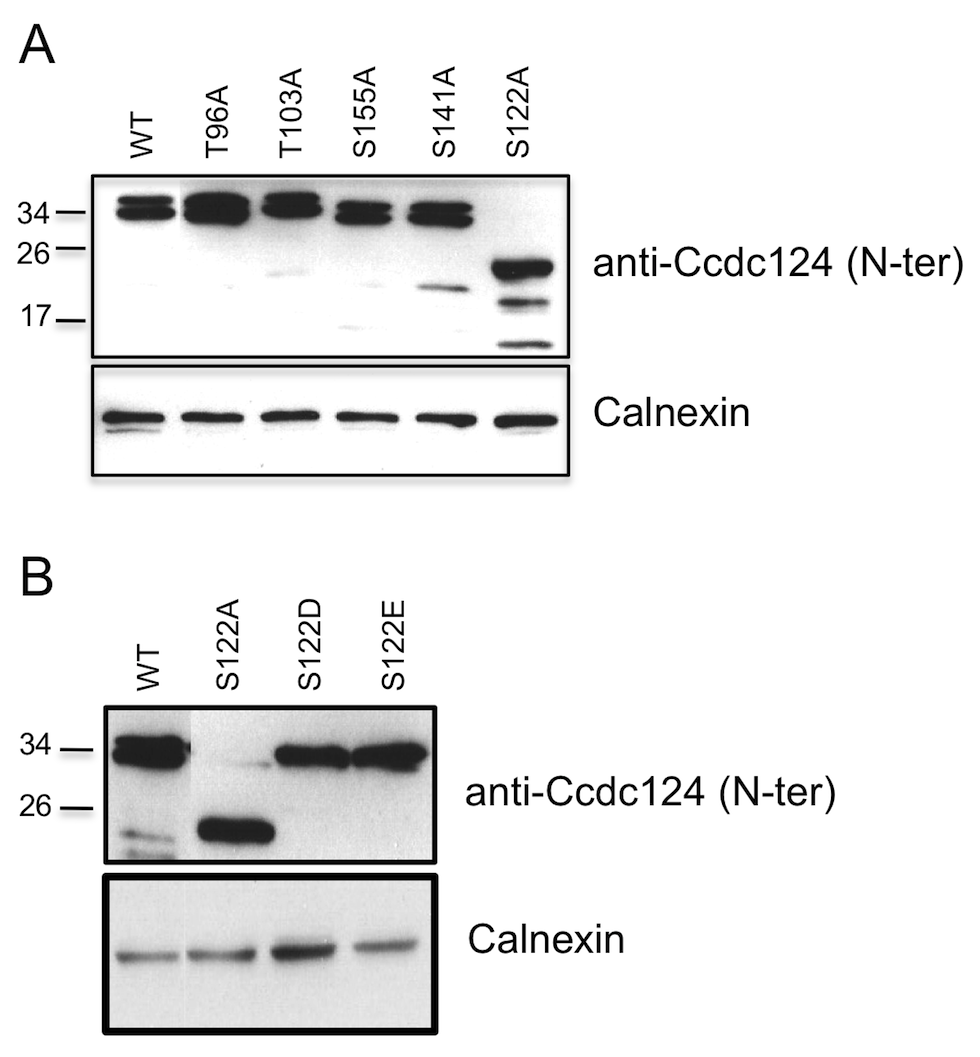

Supplement: Figure S6 — Mutating the consensus CK2 phosphorylation site Ser122 to Ala leads to compromised stability of Ccdc124. (A–B) HEK-293 cells were transfected either with HA-tagged wild-type Ccdc124 expression vector, or with similar vectors carrying indicated mutations on Figures, and stabilities of mutants proteins were monitored by immunoblots using anti-Ccdc124 antibodies recognizing the N-terminus of the protein. Only one CK2 phosphorylation consensus site (Ser122) turned out to be essential for the stability of Ccdc124 protein as S122A mutants were cleaved at their C-terminus, whereas phospho-mimicking mutations S121D, and S121E were normal in terms of Ccdc124 stability (B). Calnexin expressions were monitored as loading control. (TIFF) [file pone.0069289.s006.tiff]
