# Supplementary material for: Coiled-Coil Domain Containing Protein 124 Is a Novel Centrosome and Midbody Protein That Interacts with the Ras-Guanine Nucleotide Exchange Factor 1B and Is Involved in Cytokinesis
Source: PLoS One. 2013 Jul 19;8(7):e69289. doi: 10.1371/journal.pone.0069289 (PMC3716640; doi:10.1371/journal.pone.0069289)
Supplement: Methods S1 — (DOCX) [file pone.0069289.s007.docx]

**Supporting Materials and Methods**

***FACS Analysis***

Cell cycle status of samples was established by FACS analysis as previously described by Fabbro *et al*. [13].

***In vitro site directed mutagenesis***

Ccdc-124 Ser, Thr, Tyr putative phosphorylation site mutants were generated on the plasmid vector containing HA-linked version of Ccdc124 (pHA-Ccdc124) using the following sense and antisense primers:

T96A: (S) 5’-ACGTCCAGCAAGGTCGCCCGGGCCCAGATCGAG-3’;

(AS) 5’-CTCGATCTGGGCCCGGGCGACCTTGCTGGACGT-3’.

T103A: (S) 5’-GCCCAGATCGAGGACGCGCTGCGCCGAGACCATCAG-3’; (AS) 5’-CTGATGGTCTCGGCGCAGCGCGTCCTCGATCTGGGC-3’.

S122A: (S) 5’-GCCGAGAAAGCCAAGGCCCATCTGGAGGTGCCG-3’;

(AS) 5’-CGGCACCTCCAGATGGGCCTTGGCTTTCTCGGC-3’;

S122D: (S) 5’-GCCGAGAAAGCCAAGGACCATCTGGAGGTGCCG-3’,

(AS) 5’-CGGCACCTCCAGATGGTCCTTGGCTTTCTCGGC-3’;

S122E: (S) 5’-GCCGAGAAAGCCAAGGAGCATCTGGAGGTGCCG-3’,

(AS) 5’-CGGCACCTCCAGATGCTCCTTGGCTTTCTCGGC-3’;

S141A: (S) 5’-GTGCTGGAGGAGGGCGCCGTGGAGGCGCGCACC-3’;

(AS) 5’-GTGCTGGAGGAGGGCGCCGTGGAGGCGCGCACC-3’.

S155A: (S) 5’-GCCATTGCAGTGCTCGCCGTGGCGGAGGAGGCG-3’;

(AS) 5’-CGCCTCCTCCGCCACGGCGAGCACTGCAATGGC-3’.

100 ng template DNA and 10 pmol of (S) and (AS) primers was used in mutagenesis PCR reactions which involved 5 min initial denaturation at 95°C followed by 18 cycles of 1 min 60°C, 6 min 68°C, 1 min 95°C, and the final extension step of 10 min 68°C. Then, samples were subjected to DpnI digestion and transformed into *E. coli* DH5a competent cells. The plasmids were isolated and their sequences were verified by sequencing.
